# Reports of deaths are an exaggeration: all-cause and NAA-test-conditional mortality in Germany during the SARS-CoV-2 era

**DOI:** 10.1098/rsos.221551

**Published:** 2023-08-02

**Authors:** R. Rockenfeller, M. Günther, F. Mörl

**Affiliations:** ^1^ Mathematical Institute, University of Koblenz, Koblenz, Germany; ^2^ Computational Biophysics and Biorobotics, Institute for Modelling and Simulation of Biomechanical Systems, University of Stuttgart, Stuttgart, Germany; ^3^ Friedrich–Schiller–Universität, Jena, Germany; ^4^ Forschungsgesellschaft für Angewandte Systemsicherheit und Arbeitsmedizin mbH, AG Biomechanik and Ergonomie, Erfurt, Germany

**Keywords:** excess deaths, age cohorts, prognosis model

## Abstract

Counts of SARS-CoV-2-related deaths have been key numbers for justifying severe political, social and economical measures imposed by authorities world-wide. A particular focus thereby was the concomitant excess mortality (EM), i.e. fatalities above the expected all-cause mortality (AM). Recent studies, *inter alia* by the WHO, estimated the SARS-CoV-2-related EM in Germany between 2020 and 2021 as high as 200 000. In this study, we attempt to scrutinize these numbers by putting them into the context of German AM since the year 2000. We propose two straightforward, age-cohort-dependent models to estimate German AM for the ‘Corona pandemic’ years, as well as the corresponding flu seasons, out of historic data. For Germany, we find overall negative EM of about −18 500 persons for the year 2020, and a minor positive EM of about 7000 for 2021, unveiling that officially reported EM counts are an exaggeration. In 2022, the EM count is about 41 200. Further, based on NAA-test-positive related death counts, we are able to estimate how many Germans have died *due to* rather than *with* CoViD-19; an analysis not provided by the appropriate authority, the RKI. Through 2020 and 2021 combined, our due estimate is at no more than 59 500. Varying NAA test strategies heavily obscured SARS-CoV-2-related EM, particularly within the second year of the proclaimed pandemic. We compensated changes in test strategies by assuming that age-cohort-specific NAA-conditional mortality rates during the first pandemic year reflected SARS-CoV-2-characteristic constants.

## 1. Introduction

A *mortality rate* is the number (*count*) of how many people have died in a specific country within a defined time interval, *normalized* to the whole number of inhabitants (population size). Counts may also be done, for example, only within age cohorts, and the corresponding rates are then usually normalized to the cohort size. If counts do not ask for any death condition then all-cause mortality (AM) is registered. The balance of *all-cause* mortality rate (AMR) and birth rate determines whether there is overall growth or loss in the population. Across the world, the most usual and wide-spread way of documenting an all-cause mortality count (AMC) and an AMR by a state authority is to report them annually. Depending on the geographical, climatic and socio-economic situation, AMRs differ between countries [1]. In Europe and in the USA, for example, the annual AMRs are a little bit higher than one percent per year. Unsurprisingly, the AMRs in European and North American societies are mainly determined by the high probabilities of the eldest passing away [2]. The world-wide AMR has been generally decreasing during the last hundred years (e.g. [3–6]).

From the nineteenth century until now, the number of deaths due to infectious diseases were documented in more developed countries. Infectious diseases have a distinguishable impact on mortality. In Spain, from 1980 until 2011 as an example, mortality related to infectious diseases accounted for almost 15% of the AM [7], even though this percentage was decreasing during that period. As another example, data from cemeteries document a cholera epidemic in Brazil from 1855 to 1856, which resulted in as much as 70% more death counts in slaves than in free people, whereas the disease-specific mortality rate was identical [8]. Furthermore, people of different origin showed different mortality rates.

The influenza pandemic between 1918 and 1921 significantly increased the mortality in European countries [9] as well as in Arizona [10]. During that period, increased mortality occurred within few weeks, and mainly people between 20 and 50 years of age were affected by this influenza pandemic. A socio-economic phenomenon could be identified in Estonia after the year 1989, where the mortality in poorly educated subjects increased, while well-educated subjects had a constant mortality [11]. Taking the 2010–2011 Cholera epidemic in Haiti [12] as an example for the dynamics of infectious diseases, the AMR increased by about a factor of almost three over ten weeks; after that, the AMR decreased to values lower than usual. The disease-related mortality rates differed regionally.

In summary, a typical epidemic mortality pattern can be observed: initially, the mortality increases by a factor of up to ten within a few weeks; after this period, there is usually mortality below average for several weeks. Accounting mortality over a whole year in extreme situations like the influenza pandemic one hundred years ago, the annual AMR can be as high as two-thirds [9,10] above the regular values (an annual excess of 66%). As a rule however, the all-cause and all-age mortality is dominated by specific disease sensitivities of some cohorts within the population, which may be old people or subjects with specific (health) conditions [2,10]. Moreover, socio-economic factors can have a strong longer-term impact on mortality rates [11].

When proclaiming a SARS-CoV-2 pandemic in Europe in March 2020, the leading politicians of almost all northern, central, western and southern European countries argued the same way, namely, that CoViD-19 (or ‘C19’) would presumably kill more people than usual. In other words, a significant increase in mortality was to be expected. Even in late May 2021 [13], German media and government officials in unison referred to weekly 1300 deaths related to C19. This would have indeed been an excess mortality (EM) of 7% in Germany, given that, in the calendar week 19 of 2021 (10–16 May), 18 534 (with 95% CI [16 811; 20 258]) all-cause fatalities per week were the regularly expected AM background, according to our (exponential) model-based AM calculations in §2.3 below in this paper. However, summing daily AMCs according to the data collected by Statistisches Bundesamt (Destatis; the German bureau for statistics) [14] resulted in exactly 18 579 persons who had died in Germany in this very calendar week (19) just before the above-cited press conference. Thus, the ‘awfully high number’ of 1300 ‘C19 deaths’, which were announced with great media impetus by both the German minister of health and the head of the Robert-Koch-Institut (RKI; the German centre for disease control), occurred in a week showing mortality *perfectly matching* expectations. In Germany, thus, simply and exactly *nothing exceptional* was going on epidemiology-wise, nothing of a deadly epidemic scenario played out at all. In any case, we had already wondered in the earliest ‘pandemic’ days, and nowadays even much more, where the vast majority of the media, in unison with authorities and their scientific consultants, took such large numbers from. More explicitly, we wondered why the German authorities refused to provide context to the public in order to assess the obtrusively proclaimed number of SARS-CoV-2-related (C19) deaths. For the commonality, these numbers looked like (significant) EM.

In March 2022, the World Health Organization (WHO) published an update of SARS-CoV-2-related (‘associated with COVID-19’) EM in Germany during the years 2020 and 2021, with a 2 years total of 194 988 (see [15]). They had calculated these numbers using a Bayesian sampling model with spline-based seasonal variation [16,17]. Therewith, the C19 deaths counted by German authorities had even been claimed to under-estimate the actual EM. Later, two WHO-endorsed model upgrades [15,18] provided a reduction to a 2 years total of 122 000 excess deaths, i.e. close to the German authorities’ C19 death counts. The currently available WHO dataset [19], however, now contains the excess number 101 505 [19], which is even less than the RKI-claimed [20] 115 537 C19 deaths and does not appear in any of the aforementioned WHO-endorsed publications.

With using only such data for analysis that have been published by German authorities themselves, we now aim in this study at providing estimates of both all-cause and SARS-CoV-2-related mortality, including excess numbers in both cases, for eventually providing a well-founded, sound and reliable final assessment of the epidemiological impact of C19 in Germany between 2020 and 2022. Particularly, we provide hitherto missing context for SARS-CoV-2-related mortality numbers, both annually and flu-seasonally. In brief, we suggest to calculate, in each age cohort separately, a SARS-CoV-2-related excess count (due to C19) by subtracting the cohort’s expected all-cause estimate from all the SARS-CoV-2-related (with C19) fatalities counted therein.

## 2. Methods

### 2.1. Terminology and mathematical symbols

We start our considerations with a synopsis of the terminology used, and some explanations that also include abbreviations as well as mathematical symbols introduced.

A ‘case’ of SARS-CoV-2 infection is indicated per legal definition, in Germany as commonly around the world, by a person being tested positively using a nucleic acid amplification (NAA) test [21] applied to sputum taken from the person’s throat or fluid from the nasopharynx. In Germany, to the best of our knowledge, an NAA test is mostly a laboratory-conducted PCR [22], and only in rare cases a so-called ‘point-of-care’ (PoC) test. This is, although there are strong indications of a PCR test not being infection-indicative at all (e.g. [23]), basically due to the fact that a NAA test checks for virus material in the mucous membrane rather than for indicators of responses by the immune system [24–26]. The German authorities, like most authorities around the world, yet relate a death to SARS-CoV-2 if the deceased had received such a positive NAA test result and (presumably, since criteria are not transparently communicated to the public, at least in Germany) had shown some clinical symptoms of C19 before passing away. We use the term *C19 death* as an abbreviation of such a putatively SARS-CoV-2-related, NAA-test-conditional (C19) death.

In the following brief overview, we introduce all essential abbreviations, despite some doubling with the introduction:

— AM for ‘all-cause mortality’, i.e. deaths occurring *due to any cause*.
— AMC for an ‘AM count’, i.e. the sum of registered cases of deceased persons within a given time interval (i.e. *per time unit*), whether a day, week, year, or season; mathematical symbol for any AMC: *D*_AM_ or, if referring to a particular cohort, *D*_AM,coh_.
— AMR for ‘AM rate’, i.e. the ratio of an AMC within a group (e.g. age cohort or specific population) to the sum of all group members (e.g. cohort size *N*_coh_ or number of inhabitants *N*_pop_); mathematical symbol for any AMR: *r*_AM_ := *D*_AM_/*N*_pop_. If the AMR of a specific time interval (e.g. flu season; *r*_AM,*f*_) is scaled to a full year AMR, it is marked with an asterisk (e.g. $r_{\mathrm{AM},f}^{*}$).
— PF for ‘positive-NAA-test-conditional fatality’, i.e. deaths occurring *with* the deceased persons having received a positive NAA test result some time before passing away; in the following, ‘positive-NAA-test-conditional’ is further contracted to ‘NAA-conditional’.
— PFC for a ‘PF count’, i.e. the sum of registered cases of NAA-conditionally deceased persons within a given time interval; mathematical symbol for any PFC: *D*_PF_.
— PFR for ‘PF rate’, i.e. the ratio of a PFC within a group (e.g. age cohort or specific population) to the sum of all group members (e.g. NAA_pos_); mathematical symbol for any PFR: *r*_PF_ := *D*_PF_/NAA_pos_.
— EM for ‘excess mortality’, i.e. observed mortality (whether all-cause or any conditional) beyond expected mortality (usually, like in this study, AM).
— EMC for ‘EM count’, i.e. the difference between an observed mortality count (in this study, AMC or PFC) and the expected AMC.
— SMR for ‘standardized mortality ratio’, i.e. the ratio, within a group of persons fulfilling specific conditions (e.g. NAA-positive or with a disease), of an observed mortality count (in this study, PFC) and the expected one (usually, like in this study, AMC); mathematical symbol for the SMR of NAA-positive persons: *σ*_PF_ = *D*_PF_/*D*_AM/PF_, with *D*_AM|PF_ being the number of all-cause deaths *expected among the group members*.
— The mathematical symbol *n*_tpp_ represents the number of NAA *tests per person* in a given time interval.

### 2.2. Consulted datasets

Here is a concise synopsis of the German data sources that we based the present analysis and model development on:

— Earlier (2000–2015) all-cause daily and weekly death counts (broken down by age cohorts) were taken from the Destatis webpage at [27].
— Later (2016–2023) all-cause daily and weekly death counts (broken down by age cohorts) were taken from the Destatis webpage at [14].
— Weekly C19 death counts (broken down by age cohorts) were taken from the RKI webpage at [20].
— Counts of weekly SARS-CoV-2 ‘cases’ (i.e. numbers of positive NAA tests, broken down by age cohorts; for person counts, we relate to §2.5 and appendix A) were taken from the RKI webpage at [28].
— The demographic (age cohort) distribution until 2020 (based on the 2011 census) and its presumable distribution onwards (based on the most plausible scenario, the default model variant V1) were taken from the Destatis webpage at [29].
— Estimated counts of seasonal flu deaths were taken from the 2018 influenza report by the RKI [30].

To make all above datasets comparable, weekly time resolution was chosen as a default for both death counts and putative SARS-CoV-2 ‘cases’ (in fact, counts of NAA-positive persons), and thus for quantifying AMCs and PFCs (i.e. counts per week). For allowing specific comparisons or statements, data were occasionally contracted to annual values, and to address and solve some subtle modelling issues, we sometimes went back to daily resolution, which is then pointed out explicitly. The age cohorts were categorized in eight groups in terms of the unit *year*: 0–29, 30–39, 40–49, 50–59, 60–69, 70–79, 80–89, 90+. This grouping constitutes the finest common resolution of the above datasets.

Note that it makes a difference whether death counts are reported by Destatis on a weekly or daily basis. According to an ISO standard [31], a German statistical year consists of either 52 weeks (i.e. only 364 days taken into account) or 53 weeks (371 days), as is well reflected by the daily death counts (black spots in figures 4 and 5) differing from weekly ones (orange squares in figures 4 and 5). Accordingly, every 5 or 6 years, when a 371-day years is inserted in the official statistical data presentation, the weekly counts show a leap artefact: when compared with a regular calendar year containing 365 days (or 366 in a leap year), the missing 1-day contributions (presently in Germany: about 2800 fatalities) of the preceding statistical 364-day years are showing up due to summation over 7 days more than in the years before.

### 2.3. Two models for calculating expected AMCs, and hence EMCs, for whole years and flu seasons

**Figure 1.**
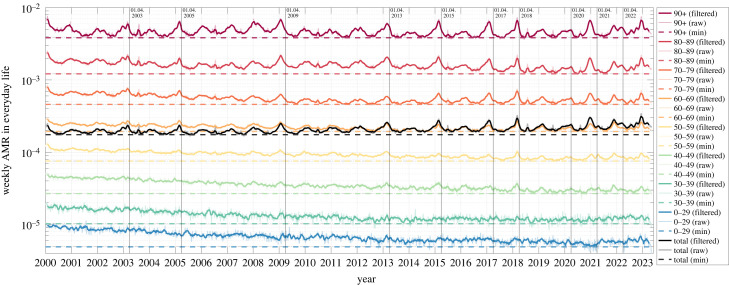
Weekly German AMR time courses between 2000 and 2022. Colours represent different age-cohort-specific as well as total (black) AMRs. Note that the raw data (dotted lines) have been smoothed (solid lines) here for better depiction by a moving average filter of a width of five weeks. The absolute minimum for each (filtered) weekly cohort AMR is indicated by a horizontal coloured dashed line in order to better observe temporal trends (figure 4 and 5). Vertical solid lines are located at the first of April, i.e. the approximate end of prominent flu seasons, i.e. for 2003, 2005, 2009, 2013, 2015, 2017, 2018, 2020, 2021 and 2022; cf. table 2.

**Figure 2.**
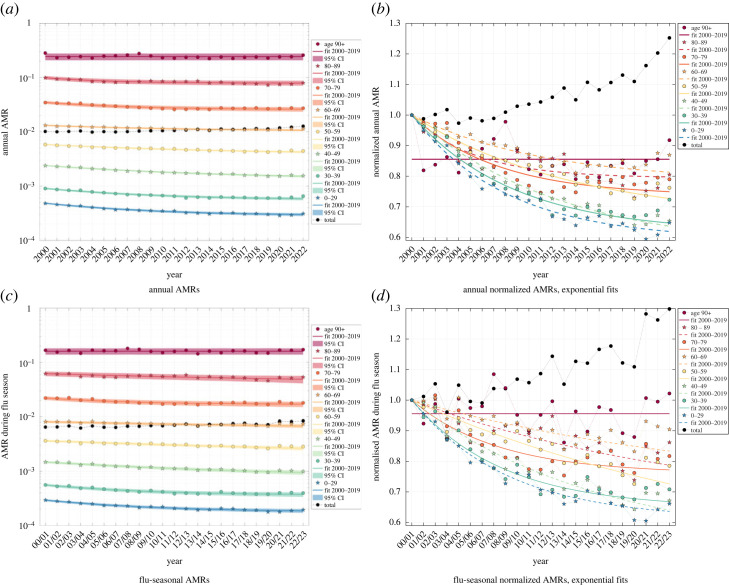
German annual (top) and flu-seasonal (bottom) AMR per age cohort from 2000–2021 and 2000/01–2021/22, respectively. The left panel contains the absolute AMR values together with exponential-fit CIs; in the right panel, for a better depiction, all AMR values are normalized to the year 2000 (season 00/01) reference value, together with the exponential point estimate. See appendix B for details on the parameter values. (*a*) Annual AMRs. (*b*) Annual normalized AMRs, exponential fits. (*c*) Flu-seasonal AMRs. (*d*) Flu-seasonal normalized AMRs, exponential fits.

When looking at the time courses of age-cohort-specific weekly AMR values *r*_AM,coh_ = *D*_AM,coh_/*N*_coh_ during the 20 years from 2000 until 2019 (figure 1), we see that they were all tendentiously decreasing, bar fluctuations in the observed data, from the early 2000s until around 2014, from which on they seem to saturate to near constant values. In our present analysis, we restricted ourselves to estimate expected *annual* AMC values (figure 2*a*, 52 calendar weeks, i.e. 364 days) as well as *flu-seasonal* ones (figure 2*c*, 33 calendar weeks from week 40 to next 20 [30], i.e. 231 days). For all of these estimated annual (figure 2*a*) and flu-seasonal (figure 2*c*) AM data points, we give the 90% confidence intervals (CIs: ± 1.645 s.d.), which account for the fluctuations in the weekly observed data (figure 1).

Here, we propose two plain AMC models for estimating *expected* (regular) AMC values in Germany, generally for an arbitrary time interval within a year. In fact, each expected-AMC model simply consists of the set of all cohorts’ AMR functions, which depend on just the observed (weekly if not daily) cohorts’ AMCs in the selected time interval within a year, the cohort sizes *N*_coh_, and the time period chosen for fitting (here, the years 2000–2019). In the first, simpler AMR model (termed ‘constant’), each cohort’s expected AMR value is a constant, the mean value over 6 years (2014–2019, see below). In the second, slightly more elaborate AMR model (termed ‘exponential’), each cohort’s expected AMR was assumed to follow an exponential course with time. The function parameters are calculated by demanding this AMR function to best fit over the chosen period to the observed (time) course of AMR data points of which each is an arithmetic mean value across the selected interval within a year (here, either the year itself or a flu season). The AMR functions can be used (i) to interpolate (estimate) potentially missing AMR data points within the fitting period, (ii) to estimate expected AMR (and thus AMC) values at any data point within the chosen fitting period or (iii) to extrapolate (prognosticate) expected AMR (and thus AMC) data points at times beyond 2019, the latter two being the model applications within this study.

**Figure 3.**
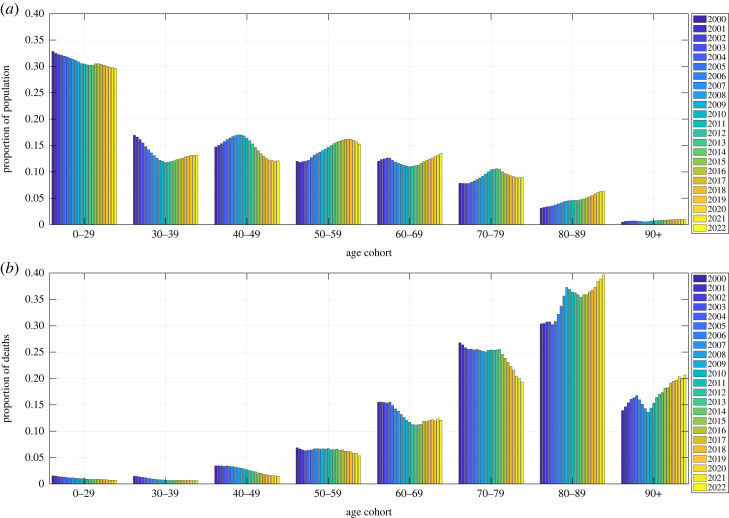
Demographic distribution of the German population’s relative proportions (in per cent), between the years 2000 and 2022, of age cohorts (*a*) and death counts (*b*). In 2022, the officially stated number of German inhabitants was about *N*_pop_ = 84 million [32].

In the simpler, ‘constant’ model, we take advantage of the aforementioned saturation of the German AMR between 2014 and 2019 to calculate a mean annual AMR value for each cohort. We do so by fitting a constant function in a least-squares sense to the six data points, provided by the *fit* routine of the ‘Curve Fitting Toolbox’ in MatLab (version R2022b). Further using the *predint* routine, we calculate a 90% CI for the AMR in each age cohort. With this, we prognosticate the expected total German AMCs for the years 2020–2022 by calculating a weighted sum of the obtained (time-constant) AMR values (and intervals), with each cohort’s size *N*_coh_ taken from the observed (time-dependent) demographic distribution in the prognosis year. Note that the total CI radius is obtained by taking the square-root of the sum of the squared weighted-age-cohort-CI radii, according to the variance sum theorem. Accordingly, in the constant model for expected AM, the course of the total population’s AMC over both the fitting and prognosis time periods is solely due to shifts of the demographic distribution with time (figure 3). This method is comparable to the calculations provided by Destatis, where the weekly AMR medians of the four preceding years in each age cohort are weighted with the population in each cohort of the current year [33].

In the more elaborate, exponential model, we take all available ‘pre-pandemic’ years 2000–2019, again conducting a fitting procedure, to determine best fitting parameters (two for each cohort: *a*_coh_ and *b*_coh_) of the exponential AMR function

2.1 $${\tilde{r}}_{\mathrm{AM},\mathrm{coh}}(\mathrm{year})=(1-b_{\mathrm{coh}})\cdot \exp(-a_{\mathrm{coh}}\cdot \mathrm{year})+b_{\mathrm{coh}}$$

to the year-resolved *normalized* AMR data points of each age cohort separately, ${\tilde{r}}_{\mathrm{AM},\mathrm{coh}}=r_{\mathrm{AM},\mathrm{coh}}/r_{\mathrm{AM},\mathrm{coh},\mathrm{ref}}$. The normalization parameters are the reference values *r*_AM,coh,ref_ = *r*_AM,coh_(2000), i.e. the cohorts’ AMR values observed in 2000. We then prognosticate the expected AMCs for the years 2020–2022 by extrapolating these exponential AMR model fits and performing the same age-cohort AMR weighting in each prognosis year as for the constant AMR model.

Note that we generally summed 52 weekly observed AMC values in one year to calculate the annual AMC values, which serve as the input for fitting the AMR function parameters. Thus, the parameters of our expected AMC models represent exactly 52 weeks, i.e. a 364-day statistical year [31]. Therefore, any AMC model output is scaled by the factor $\frac{365}{364}$ to calculate an AMC estimation of a regular year, and by $\frac{366}{364}$ in a leap year.

The same model calculations can be easily applied to estimate expected AMC values of any time interval within a year. Here, we restricted ourselves to estimate expected AMC values for only one further, specifically selected interval within a year, namely, the ‘flu season’. Accordingly, the weekly (or daily if available) observed AMC values in a year have to be contracted by summation over only the interval of a flu season. We chose a fixed length of the latter by adopting its RKI definition [30, p. 13,17], namely, the interval from (inclusive) calendar week 40 in 1 year to (likewise inclusive) 20 in the subsequent one, and thus a flu season comprising 33 weeks, i.e. exactly 231 days. After contraction of the AMC values in each of the flu seasons 2000/2001–2019/2020 (short form: 2000/01–2019/20), the fitted exponential model parameter values (i.e. those of the cohorts’ AMR functions *r*_AM,coh_(year) from equation (2.1)), and thus the cohorts’ expected AMC data points of interest, are specific for flu seasons.

This method can be generally applied: a model for calculating, in a given population, expected AMC data points can be easily constructed for *any specific* time interval shorter than a year, not only a flu season. Within each cohort and at yearly resolution, just the cohort’s weekly (or daily if available) AMC values observed have to be summed over the chosen time interval. Subsequently, the fitting procedure provides parameter values (for either the cohort’s exponential function or simply a constant, the mean of the interval sums taken into account) that represent the (smoothed) course of the cohort’s expected AMC data points over the whole fitting period.

### 2.4. PFR values from registered SARS-CoV-2 ‘cases’ and C19 death counts: solving the problems of multiple tests per person and time delays

While displaying PFR values in 2020 and 2021, we noticed extremely low PFRs from late 2021 on, when compared with the first-year SARS-CoV-2 infection period from May 2020 until summer 2021 (see appendix A, table 7 for details). Our interpretation of these massively dropping PFR values is that, from some instant in late 2021 on, there has been an evidently inconsistent relation between official ‘case’ counts (raw NAA_pos_, number of positive *tests*) and the number of positively tested *persons*. However, to calculate a meaningful PFR, the number of persons having died (*D*_PF_) must be divided by the number of *persons* tested positive (NAA_pos_ processed to represent *persons* rather than positive *tests*). In other words: since late summer 2021, the officially published (raw) NAA_pos_ (and thus PFR) values cannot be reasonably interpreted as representing positively tested persons without additional processing. Fortunately, such processing, which implies a re-interpretation of what NAA_pos_ counts mean, proved *possible*: we assume that the PFR values *until* summer 2021 are *disease characteristics*, which have been consistently determined due to a person-to-test ratio being nearly one-to-one in the first year of the ‘Corona pandemic’, i.e. from May 2020 to May 2021. With this, we re-calculated the raw NAA_pos_ values for the subsequent, second year of the ‘Corona pandemic’, i.e. May 2021 to May 2022, and its sub-period, the flu season 2021/22. We did so by multiplying the raw NAA_pos,May21/22_ values between May 2021 and May 2022 with the factor *n*_tpp_ = *r*_PF,May21to22_/*r*_PF,May20to21_, and accordingly the raw NAA_pos,flu21/22_ values for the flu season 2021/22 with *n*_tpp,flu_ = *r*_PF,flu21/22_/*r*_PF,flu20/21_. The results of this NAA_pos_ post-processing are presented in §3.3.

Another observation brought to light that reporting PFR values by dividing the number of C19 deaths per week (*D*_PF_) by NAA-positive ‘cases’ per the *same* week (NAA_pos_) is not recommended for two reasons. First, the number of NAA tests can well differ greatly by the week, so there is significant scatter along week-resolved time courses. Second, from receiving the positive test result (or its date of registration) to the actually corresponding death event, there is usually a time delay of several days or even weeks [34]. Here, we show that taking raw PFR values from an unprocessed calculation of a same-week ratio overestimates, on average, the PFR values that arise when comparing death counts to the number of positive test results from days ago. Therefore, our PFR analysis started with finding realistic delay values between the courses of NAA-positive ‘case’ counts and PFCs, one value for each age cohort. In doing so, we only relied on courses until the middle of 2021, because later (raw) NAA-positive ‘case’ counts turned out to be unreliable (see above). We determined these delay values in a straightforward way by systematical time shifting of the two datasets relative to each other, with covering the range from May 2020 to May 2021. To this end, in each cohort, (i) a (pseudo-)daily resolution was generated by applying cubic-spline interpolation to the courses of both the PFC values *D*_PF_ and the counts of NAA-positive ‘cases’ NAA_pos_, all known as weekly sums; (ii) these two day-resolved courses were systematically shifted (in discrete day steps) relative to each other, and the arithmetic mean of the (daily) PFR values (the ratio *r*_PF_ = *D*_PF_/NAA_pos_ of counts in a time unit) was calculated for each day-shift value; (iii) the very delay value with the minimal mean *r*_PF_ value for a maximally five weeks delay was identified (see figure 7; mean *r*_PF_ values in table 7).

### 2.5. C19 death counts put into context: analysing PFR(PFC) against the background of AMR(AMC)

As the final step within this study, we performed the analysis presented in §2.3 again cohort-wise and both annually, with yet the years adapted to the ‘Corona pandemic’ (i.e. across the calendar year boundaries: from May 2020 to May 2022), and for the two flu seasons in this 2-year episode. To put NAA-conditional mortality (i.e. PFC and PFR) into AM context, we applied the exponential model estimation of the age cohorts’ AMRs (§2.3, equation (2.1)), while also knowing the *NAA-conditional* cohort sizes (for each, its NAA_pos,coh_ value), to the group of Germans that have received a positive NAA test, i.e. the whole NAA-conditional sub-group of the population. Within each cohort, we contrasted the number *D*_PF,coh_ of persons in the sub-sub-group therein, i.e. C19 fatalities, with the number *D*_AM|PF,coh_ of persons who were estimated by our AMC model (equation (2.1)) to have expectably died due to any cause within the NAA-conditional sub-group of NAA_pos,coh_ persons. That is, we calculated the NAA-conditional EMC by subtracting deaths *D*_AM|PF,coh_ expected according to our AMC model from the number *D*_PF,coh_ of official C19 deaths. NAA-conditional EMCs (here, annual or flu-seasonal values) quantify *excess* deaths in the sub-group of official C19 deaths, i.e. fatalities that are in excess of what is expected due to all causes. This method of determining a conditional EMC is generally applicable to *any* conditionally selected sub-group of a population. Within the sub-group of NAA-positive persons, we suggest to identify these definite excess deaths (NAA-conditional EMCs) with those having occurred *due to* C19. The results of this analysis are presented in §3.3.

## 3. Results

### 3.1. The AMR course over 20 years: a Simpson’s paradox

In this section, we will address the beneficial representation of AMR courses over time (see §2.3) and we show that the interplay of these courses with demographic changes results in a paradoxical phenomenon. Ignoring this interplay may result in devastatingly wrong forecasts of AMCs, as is discussed later.

In order to achieve our main goal, the valid estimation of EMCs, we start here with scrutinising age-cohort-resolved AMR time courses during the 20 ‘pre-pandemic’ years, with the age cohorts being determined by the available datasets (see §2.2). The first main finding of this investigation: the trend of the cohort’s AMR time courses may well be described by plain functions versus time (§2.3). In Germany since 2000, they turned out to be well of exponentially saturating character in all but one age cohort (figure 1; more obvious when normalized to each its 2000 value: figure 2*b*). The scientific value gained by identifying such basic functions for (model-based) AMC calculations lies in (i) their potential to reflect a trace of the dynamical mechanisms behind changing AM in a population and (ii) the transparency they lend to AMR and thus AMC estimations. The latter benefit comes in a big part from the inherent character of any continuous function in time, particularly if being of exponential character: to reflect physical and biological processes, rather than possibly arbitrary, mathematical black box assumptions. Due to time-wise continuity and moreover being low-parametric (determined by only two parameters for each 20 data points; see appendix B for their values and CIs), (iii) they also have inherently a smoothing, averaging character, which is a technical advantage for using such a function-(model-)based approach to estimate AMC *base lines*.

The second main finding of this scrutiny: resolving AMC to age cohorts matters. A year-wise contraction of each age cohort’s weekly AMR time course (figure 1) into a single data point, starting with the year 2000, provided a sequence of annual AMR values (coloured bullets in figure 2*a*). Their general trend with time can be better seen by plotting each cohort’s AMR course of the annual values normalized (figure 2*b*) to its reference value (the value in 2000 or in the 2000/2001 flu season (figure 2*d*), respectively). Next, the course of the total German population’s AMR is calculated as the weighted sum over all cohorts’ normalized AMR values in a given year, with each cohort being weighted by its relative proportion (figure 3) in that year, times its respective reference value. If then both the year-resolved cohorts’ normalized AMR courses and the course of the total AMR (normalized to the sum of all cohorts’ sizes: the whole population size) are plotted over the 20 years 2000–2019 (figure 2*b*), a counterintuitive phenomenon appears in Germany: while each single cohort’s AMR tends to *decrease* over the years, in all age cohorts except the eldest whose AMR remains practically constant, the total AMR tends to *increase*, which applies consistently to both annual and flu-seasonal AMR courses.

This phenomenon is known as *Simpson’s paradox* [35–38]. It is explained by summing over sub-groups (cohorts) that differ in relative proportion (i.e. their weights, the time-dependent demographic distribution, figure 3) and effect strength (i.e. the AMR values, which are strongly age-dependent): older people die at a higher probability per time (e.g. a year, figure 2*a*, or a flu season, figure 2*c*), and, in Germany during the last about 10 years, the proportions of the 60–69, 80–90 and 90+ year-old people increased, whereas those of all people younger than 60 years decreased (figure 3). Consequently, the (average) total probability of a German of whatever age to die within a year has increased, although each German below 90 years has gained more average life time during the twenty ‘pre-pandemic’ years until 2020.

Note first that the exponential fits (according to equation (2.1)) in the 90+ cohort did not yield a meaningful result (no calculation of CIs was possible), neither for the annual values in 2000–2019 nor for the corresponding flu-seasonal values; it was therefore substituted by simply fitting a *constant* for the 2000–2019 courses of each the annual and the flu-seasonal normalized AMR values (figure 2*b*,*d*).

Note second, again, that only the data points between 2000 and 2019 (or flu seasons 2000/01–2019/20, respectively) have been used to calculate the fit parameters (separate for the years and the flu seasons). Thus, the line parts in figure 2*b* and figure 2*d* that pass the last three data points are extrapolations (prognoses) by the exponential fit functions (equation (2.1)). The overall graphs are the graphical representations of the cohorts’ *expected* AMR courses according to the exponential fit model, i.e. a smoothed (when compared with the fluctuating values observed), model-based (exponential fit) reference for estimating EMCs. Not visualized here is the simplest reference model, the ‘constant fit’, solely consisting of horizontal fit lines of which each runs through the arithmetic mean of one cohort’s AMR values observed in the last six intervals (years or flu seasons) before 2020, i.e. a constant AMR value assigned to each cohort.

Note third that, according to figure 1, nothing else than ‘AM as usual’ occurred in Germany in 2020 and 2021. Our assessment of entirely non-exceptional AMCs and all-cause EMCs in Germany during the SARS-CoV-2 era (2020 and 2021), be they assessed annually or during the flu seasons, is quantitatively substantiated in §3.2 (tables 1 and 2 in particular). Hence, it has to be assumed that any model calculation showing dramatic EMC in Germany during those 2 years fell for the Simpson’s paradox and has to be carefully reconsidered regarding the shifts in the demographic distribution, i.e. changes of the age cohorts’ sizes with time; see §4.3.

**Table 1.** Counted AMC versus model estimates (last three rows: extrapolation) in Germany during the years 2000–2022; significantly deviating death counts (outside the CI) are labelled by superscript ^(**)^; population in 2022: *N*_pop_ = 84 million [32]; ‘excess’ (EMC) is counted minus model difference; for the constant model, under-mortality of −14 055 deaths in 2020 (reference: mean of 2014–2019 AMR values) correspond to −16.9 deaths per 100 000 persons, best comparable to Sweden with about −10 per 100 000 [39, fig. 2, bottom] (reference: mean of 2015–2019 AMR values); other than (short-term: 2015–2019) trend models applied to Sweden [39, fig. 2, bottom] (about +40 per 100 000), our (longer-term: 2000–2019) trend model (exponential) calculates slightly enhanced magnitude of prognosticated under-mortality: −22.1 per 100 000. As a comparison, the last column shows EMC point estimates that arose from applying the method used by the German bureau for statistics (Destatis).

year	deaths (counted)	excess deaths (exponential model)		excess deaths (constant model)		excess deaths (Destatis method)
		mean	confidence interval	mean	CI radius	
2000	838 797	17 534	19 996	—	—	—
2001	828 541	−11 265	20 912	—	—	—
2002	841 687	5 305	21 908	—	—	—
2003	853 946	22 330	22 662	—	—	—
2004	818 271	−17 831	23 531	—	—	−68 509
2005	830 227	−420	24 413	—	—	−38 433
2006	821 627	−6 344	25 378	—	—	−43 069
2007	827 155	1066	26 268	—	—	−28 514
2008	844 439	17 279	27 254	—	—	188
2009	854 544	16 009	27 863	—	—	−8843
2010	858 768	−1001	28 369	—	—	−28 907
2011	852 328	−10 098	28 355	—	—	−37 263
2012	869 582	−2 215	28 643	—	—	−21 734
2013	893 825	14 603	28 872	—	—	1206
2014	868 356	−26 833	29 771	−7705	33 339	−38 030
2015	925 200	17 144	30 555	32 882	34 391	13 618
2016	910 899	−15 963	31 662	−3081	35 731	−22 924
2017	932 263	−7445	32 791	2742	36 938	−12 150
2018	954 874	−3053	34 180	4905	38 430	461
2019	939 520	−40 458^(**)^	35 825	−34 381	40 174	−42 132
2020	985 572	−18 471	37 561	−14 055	41 975	−595
2021	1 023 687	6992	38 855	9672	43 168	22 949
2022	1 064 084	41 210^(**)^	39 423	41 968	43 444	53 879

### 3.2. Observed AMCs versus AMC-model-calculated fatalities: EMCs, annually and during flu seasons

In this section, we show that both the constant and the exponential AMR models (§2.3) yield exceptionally good fits within the intervals 2014–2019 and 2000–2019, respectively, as well as realistic prognoses for AMCs for 2020–2022. Contrary to claims by the RKI of significant, and by the WHO even of drastic EM in Germany during the SARS-CoV-2 era we even find negative EMCs (under-mortality).

**Figure 4.**
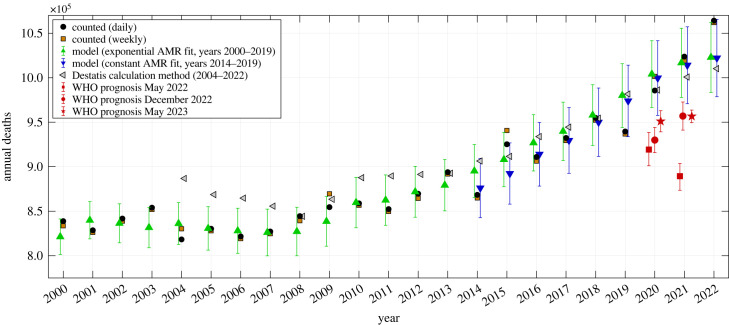
German data of counted annual deaths (derived from daily counts in black dots, from weekly counts in brown squares), versus model estimates or prognoses of expected deaths (exponential model in green upward-pointing triangles, constant model in downward-pointing blue triangles) including 90% CIs for the years 2000–2022, versus AMC calculation using the method from the German bureau for statistics (Destatis), and versus WHO’s AMC prognoses for 2020 and 2021 published in May 2022 (red squares), December 2022 (red circles) and May 2023 (red stars), including their 95% CIs.

**Figure 5.**
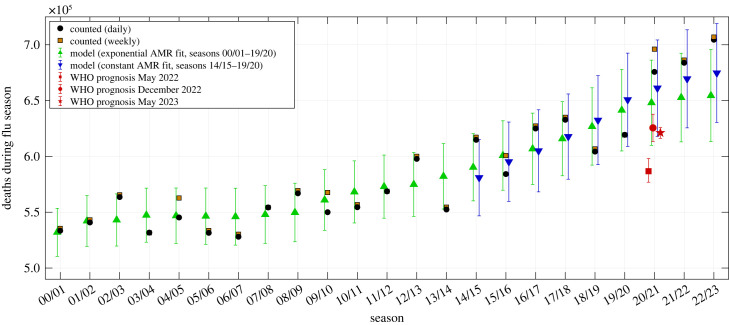
German data of counted flu-seasonal deaths (daily in black dots, weekly in brown squares), versus model estimates or prognoses of expected deaths (exponential model in green upward-pointing triangles, constant model in downward-pointing blue triangles) including 90% CIs for the seasons 2000/01 until 2022/23, and versus the WHO’s AMC prognoses for 2020/21 from May 2022 (red square), December 2022 (red circle) and May 2023 (red star), including their 95% CIs.

**Figure 7.**
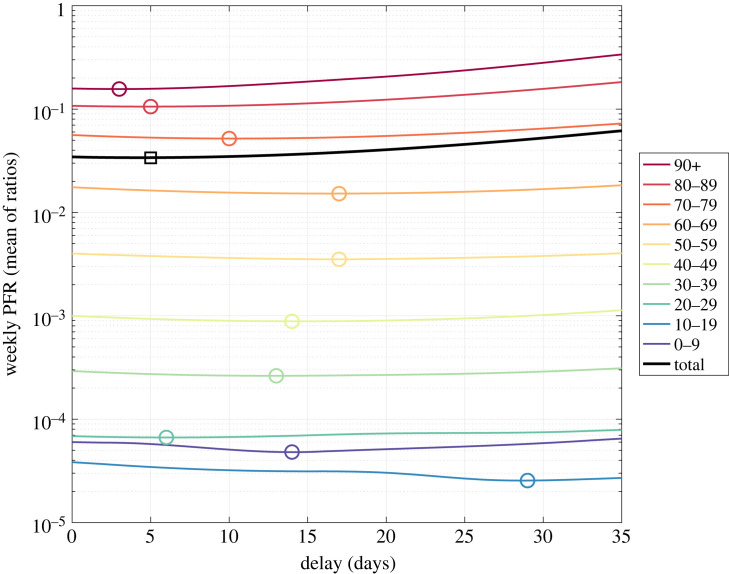
For each German age cohort, the mean of its daily PFR values (averaged across the period 05/2020 to 05/2021: sum of daily ratios *D*_PF_/NAA_pos_ divided by the number of days taken into account) versus a varied time shift of the *D*_PF_ course forward with respect to the NAA_pos_ course; both courses registered in week resolution were interpolated through cubic splines for emulating day resolution; minima are indicated as circles or a square.

In figure 4, we have plotted actually *observed* data points (counts on daily basis: black spots) and the correspondingly expected exponential model estimates (green triangles with CIs) of the total annual AMC values since the year 2000. The total-AMR-model-fit CI at any annual data point is calcuated as the weighted sum of the eight age-cohort-specific AMR model fits’ 90% CIs of that year. The annual sums of the daily observed AMCs (the only data available by day) are always within the CIs of the exponential model fit, except for the year 2019 in which significantly fewer than expected people died, and the year 2022 in which significantly more than expected people died. Accordingly, the exponential model generally well matches the observed AMCs over the whole analysed period from 2000 until today. For the flu-seasonal data in particular (figure 5), the AMCs expected from the simplest (constant) model (blue triangles with CIs) match the observed AMCs very well. All estimates by the constant model demonstrate impressively that, in Germany since 2014, the total AMC time course is dominated for the most part by shifts in the demographic distribution with time (figure 3). The differences between both models’ estimates, whether for whole years or flu seasons, are usually smaller than between an exponential model estimate and an observed AMC on a yearly basis. Note that the observed 2019/20 flu-seasonal AMC in Germany is well *below* both model estimates (figure 5), although this season contains the first SARS-CoV-2 wave of infection (start of the ‘Corona pandemic’).

For the total German population, we have listed in table 1 the exact numbers (visualized in figure 4) of observed annual AMCs (deaths) since 2000 and their corresponding differences (excess deaths: EMCs) to both the exponential and constant model estimates. The observed AMCs are generally sums over 365 days, and 366 days in a leap year. The essential numbers are the EMC values in the last three rows of tables 1, 2, and, by our analysis, we can make a clear statement: *fewer* people than expected died in 2020 (negative excess value of −18 471), and in 2021, a mild EMC of about 7000 showed up. In both ‘Corona pandemic years’, non-existing EM gives evidence of there having been no exceptional public health situation whatsoever. In contrast to our finding, the WHO calculated a total EMC of about 195 000 during 2020–2021: EMC is the difference between expected numbers, red stars with 95% CIs in figure 4, and actually observed numbers (black dots). Note that the actual AMC data points for 2020 and 2021 are unfathomable 6.8 and 18.5, respectively, standard deviations away from the WHO’s unvalidated point estimates from May 2022. For the corrected prognosis [15,18], these values are still 7.7 and 8.3 standard deviations away and for the latest dataset [19] 5.7 and 18 standard deviations. Further assessment of these models and their prognoses is given in §4.3 and appendix C.

**Table 2.** Counted AMC versus model estimates (last two rows: extrapolation) in Germany during the flu seasons 2000/01 to 2021/22; significantly deviating counts (outside the CI) are labelled by superscript ^(**)^; population in 2022: *N*_pop_ = 84 million [32]; ‘excess’ (EMC) is count minus model difference; for comparison, estimated numbers published by the RKI [30, p. 47] are given in the last column.

year	deaths (counted)	excess deaths		excess deaths		flu deaths (RKI)
		(exponential model)		(constant model)		
		mean	CI radius	mean	CI radius	
00/01	533 445	1419	21 484	—	—	—
01/02	540 826	−1359	22 670	—	—	0
02/03	563 575	20 504	23 378	—	—	8000
03/04	531 721	−15 675	24 223	—	—	0
04/05	545 301	−1557	24 770	—	—	11 700
05/06	531 520	−14 982	25 191	—	—	0
06/07	528 041	−17 992	25 507	—	—	200
07/08	554 270	6337	25 858	—	—	900
08/09	566 938	17 164	26 231	—	—	18 800
09/10	550 009	−10 965	27 131	—	—	0
10/11	554 481	−13 709	27 817	—	—	0
11/12	568 670	−4 275	28 306	—	—	2400
12/13	597 792	22 875	28 646	—	—	20 700
13/14	552 426	−29 750^(**)^	29 317	—	—	0
14/15	614 852	24 600	30 102	33 913	34 197	21 300
15/16	584 197	−16 619	31 107	−11 051	35 563	0
16/17	624 984	18 168	31 995	19 983	36 736	22 900
17/18	632 810	16 980	33 182	15 080	38 179	25 100
18/19	604 302	−22 518	34 661	−28 111	39 843	—
19/20	619 348	−22 041	36 538	−31 394	41 782	—
20/21	675 714	27 594	38 141	14 526	43 130	—
21/22	683 875	31 123	39 730	14 372	43 935	—
22/23	706 143	51 677^(**)^	41 247	31 429	44 217	—

In table 2, the exact numbers (visualized in figure 5) of observed AMCs (deaths) in Germany during the flu seasons—exactly 33 weeks, 13 at a year’s end plus the first twenty of the next: 91 plus 140 days, or 141 in a leap year—are reported. The exponential (flu-seasonal AMR fit) model output representing 33 weeks (231 days) is scaled to a leap year by the factor $\frac{232}{231}$, and, like in table 1, the differences (excess deaths: EMCs) to both the exponential and constant model estimates are also given. Like in the data for the calendar years, the essential numbers are the excess values in the last three rows. In the flu season 2020/21, an excess of 27 594 deaths can be attributed mainly to the second and third waves of putative SARS-CoV-2 infections. The excess of 30 443 deaths was even a little bit higher in the following season 2021/22. These excesses followed two seasons 2018/19 and 2019/20 with mortality below the expected value (negative excess values, i.e. ‘under-mortality’), at almost the same magnitude. This is evidently a typical pattern for seasonal flu waves: sometimes up to four flu seasons of under-mortality in a row are followed by a similar sequence of stronger flu seasons with excess values on a level of 20 000 deaths. Since the last 10 years, the magnitudes of these fluctuations seem to slightly increase, with none of the flu seasons close to the expected value occurring in between anymore. Looking back to 2000, the excess values in the two ‘Corona seasons’ have been just slightly higher than in the strongest influenza seasons of the 20 ‘pre-pandemic’ years (2002/03, 2012/13, 2014/15). The two ‘Corona seasons’ were thus not at all on exceptional mortality levels. Our rating of flu seasonal EM is reconfirmed by published RKI numbers [30, p. 47]: since season 2000/01, they specified four excess estimates above 20 000 deaths, the highest (25 100) in the season 2017/18, which may be seen as a calibration value for an EMC occurring in a significant (moderately strong yet usual) flu season in the last decade. Notably, we found a higher excess than the RKI in the season 2002/03, and there are remarkable near-matches in 2008/09, 2012/13 and 2014/15 (referring to our exponential model at least). According to our analysis, the RKI excess values in 2016/17 and 2017/18 even seem to be slight exaggerations.

Interestingly, on the one hand, even under-mortality and a very low excess can be seen in the ‘Corona pandemic’ years 2020 and 2021, respectively. But, on the other hand, one can see significant excess deaths during the flu seasons 2020/21 and 2021/22. Correspondingly, there must have been significant under-mortality in the spring and summer periods in 2020 and 2021, which compensated flu-seasonal excess within few months and can indeed be clearly observed in figure 4 in the age cohorts between 60 and 89 years. We come back to this phenomenon of compensatory under-mortality in the next section, which looks at NAA-conditional mortality.

### 3.3. Putting NAA-conditional death counts into context: PFC against AMC background

**Figure 8.**
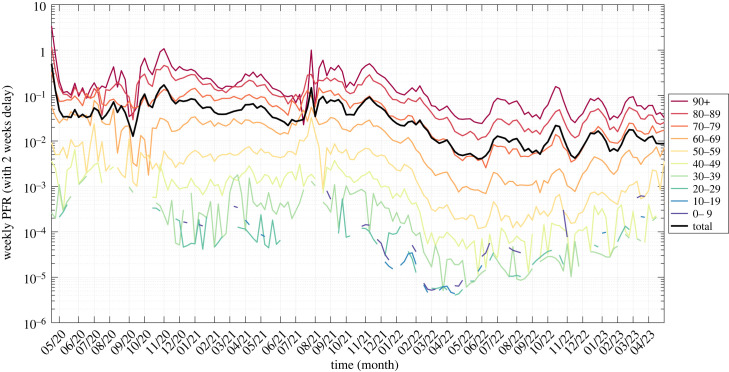
For each German age cohort, the week-resolved PFR values (*D*_PF_/NAA_pos_) in the period 05/2020 to 07/2022, generally applying a forward time shift of two weeks to *D*_PF_.

This section addresses the question ‘Have people died with or due to SARS-CoV-2?’. For this, we simply put NAA-conditional death counts (PFCs) into the context of regularly expected, all-cause death counts (AMCs), i.e. present the results of calculating NAA-conditional EMCs (according to §2.5). They are based on the processing of raw PFC data (NAA_pos_ as observed) and corresponding PFR determination, as explained in §2.4. For each age cohort, we took its AMR values (figure 2), as well as its count of NAA-positive persons (NAA_pos_ processed) and its number of fatalities proclaimed to be related to SARS-CoV-2 (*D*_PF_) two weeks later, the latter two combined into PFR values (*r*_PF_ = *D*_PF_/NAA_pos_; figure 8 and table 7).

As shown in appendix A, the (raw) number NAA_pos_ of NAA-positive tests evidently has to be re-interpreted since the middle of 2021 when compared with the start of the RKI publishing these data since early 2020: only until summer 2021, tests and persons are practically congruent, afterwards, NAA_pos_ must be processed to represent persons. All other data can be used as given above, the AMR values in particular, as they are only depending on plain, non-processed counts of all German citizens, and of all who died of any cause during the past: the plain registration of all deaths in files maintained by local authorities. Likewise, the NAA-conditional death counts *D*_PF_ seem to be consistent; in other words: the RKI criteria for proclaiming a death to be ‘related to SARS-CoV-2’ do not seem to have changed during the whole ‘Corona pandemic’ since early 2020. Such consistency can be most notably concluded from the fact that, after having applied our processing (see §2.4) to the officially published NAA_pos_ data since the middle of 2021, the post-processed, inferred relative distribution of excess (NAA-conditional minus all-cause) death counts in 2021/2022 across age cohorts, given in tables 5 and 6, are in very good proportion and accordance with those calculated without any NAA_pos_ processing in 2020/2021, given in tables 3 and 4, respectively.

**Table 3.** Calculated German NAA-conditional (C19) EMCs (excess) between May 2020 and May 2021; AMC model estimation: *D*_AM|PF_; SMR value: *σ*_PF_ = *D*_PF_/*D*_AM|PF_.

age cohort	*r*_AM_	NAA_pos_	*D*_PF_	*r*_PF_	*D*_AM|PF_	excess	*σ*_PF_
90+	0.2426	69 308	17 648	**0.2546**	16 947	701	1.04
80–89	0.07555	193 560	36 462	**0.1884**	15 466	20 996	2.36
70–79	0.02734	176 848	16 640	**0.09409**	4 665	11 975	3.57
60–69	0.01116	302 295	7 226	**0.0239**	3 278	3 948	2.20
50–59	4.363 × 10^−3^	537 282	2624	**4.884** × **10**^−**3**^	2324	300	1.13
40–49	1.541 × 10^−3^	467 046	592	**1.268** × **10**^−**3**^	727	−135	0.82
30–39	6.012 × 10^−4^	503 153	186	**3.697** × **10**^−**4**^	300	−114	0.62
0–29	2.792 × 10^−4^	1 015 378	64	**6.303** × **10**^−**5**^	309	−245	0.21
total	0.01201	3 264 870	81 442	**0.02494**	44 015	37 427	1.85

**Table 4.** Calculated German NAA-conditional (C19) EMCs (excess) during flu season 2020/21; AMC model estimation: *D*_AM|PF_; SMR value: *σ*_PF_ = *D*_PF_/*D*_AM|PF_.

age cohort	$r_{\mathrm{AM},f}^{*}$	NAA_pos_	*D*_PF_	*r*_PF_	*D*_AM|PF_	excess	*σ*_PF_
90+	0.268	68 104	17 449	**0.2562**	10 944	6505	1.59
80–89	0.08329	189 978	36 002	**0.1895**	9375	26 627	3.84
70–79	0.02998	173 305	16 358	**0.09439**	2959	13 399	5.53
60–69	0.01202	296 718	7080	**0.02386**	2035	5 045	3.48
50–59	4.671 × 10^−3^	525 226	2558	**4.870** × **10**^−**3**^	1403	1155	1.82
40–49	1.631 × 10^−3^	452 767	575	**1.270** × **10**^−**3**^	434	141	1.33
30–39	6.298 × 10^−3^	486 836	180	**3.697** × **10**^−**4**^	183	−3	0.98
0–29	2.809 × 10^−3^	976 642	62	**6.348** × **10**^−**5**^	184	−122	0.34
total	0.01321	3 169 576	80 264	**0.02532**	27 517	52 747	2.92

**Table 5.** Calculated German NAA-conditional (C19) EMCs (excess) between May 2021 and May 2022; multiple NAA tests: *n*_tpp_; AMC model estimation: *D*_AM|PF_; SMR value: *σ*_PF_ = *D*_PF_/*D*_AM|PF_. Bold numbers are due to *r*_PF_ being fixed to the value between May 2020 and May 2021 (table 3).

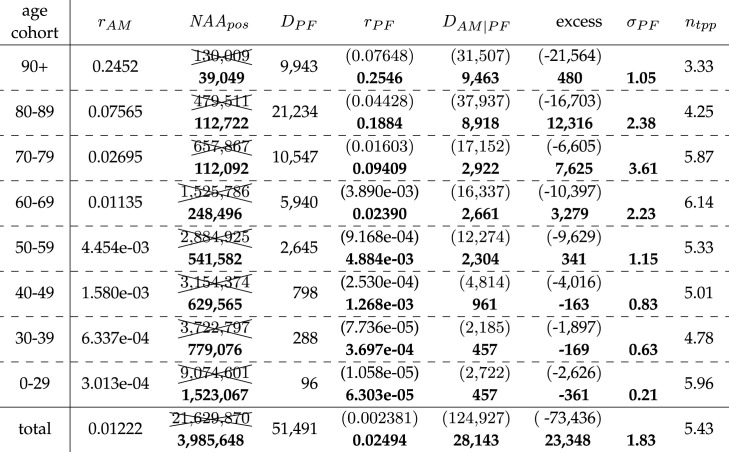

**Table 6.** Calculated German NAA-conditional (C19) EMCs (excess) during flu season 2021/22; multiple NAA tests: *n*_tpp_; AMC model estimation: *D*_AM|PF_; SMR value: *σ*_PF_ = *D*_PF_/*D*_AM|PF_. Bold numbers are due to *r*_PF_ being fixed to the value during the flu season 2020/21 (table 4).

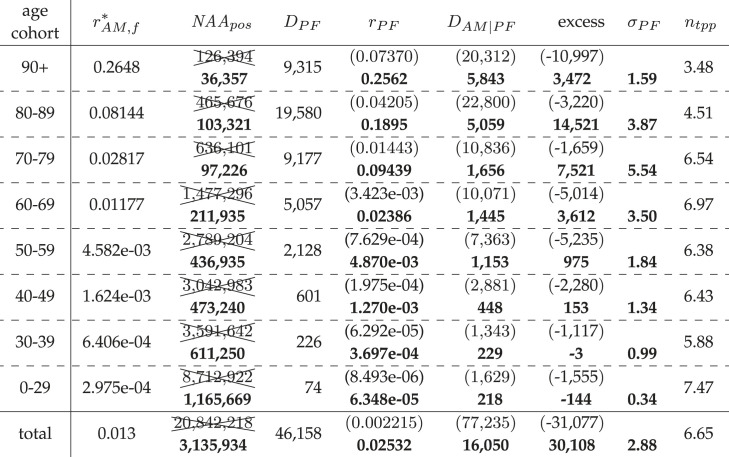

The PFR values for May 2020 until May 2021, practically identical to those in the corresponding flu season and taken as fixed disease characteristics, are presented in bold font in tables 3 and 4. The result of the NAA_pos_ processing can be seen in tables 5 and 6 in which we have crossed out the raw NAA_pos_ numbers, put all values depending thereon in round brackets, and presented all processed results also in bold font. The ratios *n*_tpp_ and *n*_tpp,flu_, which we would interpret as the number of multiple NAA tests per person within a year or flu season, respectively, are given in the last columns of tables 5 and 6, respectively. The advantage of analysing the period of two whole-year intervals between May 2020 and May 2022, respectively, instead of the two calendar years 2020 and 2021 as in §3.2, is that a May-to-May interval better distinguishes the ‘Corona pandemic’ situation (its ‘waves’). Moreover, the mortality outside a flu season can be easily seen from the differences between a whole May-to-May year, ending at calendar week 20, and its entire ‘flu season’ included (e.g. compare tables 3 and 4).

When looking at the ‘Corona pandemic’, first-year period, May 2020 until May 2021, we can see by comparing table 3 with table 4 that there were practically vanishing infection dynamics from late May 2020 until end of September 2020, since all numbers in the whole-year period May 2020–May 2021 only minutely differ from the corresponding flu season. The main result is found in the last columns of tables 3 and 4. For each cohort, the calculated deaths in excess (NAA-conditional EMC) of what is expected according to AMR. These EMC values are what *might eventually* be related to SARS-CoV-2 in an epidemiological sense. The total EMC value in the NAA-conditional sub-group in Germany from May 2020 until May 2021 was 37 427, which contrasts to the RKI officially declared 81 433 ‘C19 deaths’. Adapting the RKI nomenclature of deaths ‘due to flu’ (‘Todesfälle durch Influenza’ [30, table 3]; see also table 2, last column) to the context of SARS-CoV-2, this total EMC value of 37 427 can be seen as the number of persons who have died ‘due to C19’, whereas one might say that 81 442 persons have died ‘with C19’. Notably, the total NAA-conditional EMC was clearly higher in the corresponding flu season 2020/21, namely, 52 747 deaths. This gives clear evidence that many of the persons who deceased in the flu season had only few months to live anyway, as an under-mortality must have occurred, even in the NAA-conditional sub-group of Germans, in both the summer periods 2020 and 2021. Further, from May 2021 until May 2022, the EMC value 23 348 was lower than the EMC from May 2020 until May 2021 (37 427, table 5) and also lower than the EMC of the corresponding flu season 2021/22 (30 108, table 6). That the NAA-conditional sub-group is definitely *not* representative of the whole German population, and therefore only tells part of the total German infection dynamics in the ‘Corona pandemic’ first-year period, becomes evident from the total unconditional (i.e. all-cause) flu-seasonal EMC (27 594 with exponential model, or even just 14 526 with constant model; see table 2) being clearly lower than the total flu-seasonal EMC in the NAA-conditional sub-group (52 747). In the second ‘Corona pandemic’ flu season (2021/22), however, NAA-conditional (30 108) and all-cause (31 123) EMCs match well.

As a side note of having the raw counts of positive NAA tests post-processed in the intervals May 2021–May 2022 and flu season 2021/22, we can eventually interpret the resulting, processed NAA_pos_ values (bold numbers in tables 5 and 6, respectively) once again as the numbers of positively tested persons; the corresponding numbers *n*_tpp_ and *n*_tpp,flu_ have then gained the meaning of the average number of NAA tests per person and year or flu season, respectively; their moderately cohort-dependent values are reported in the last columns of tables 5 and 6. The annual *n*_tpp_ values range from 3.33 to 6.14, and *n*_tpp,flu_ from 3.48 to 7.47 in the flu season. It seems that the test frequency has been raised most notably in the flu season 2021/22, particularly in the youngest.

We recapitulate: according to our model-based estimations, 37 427 Germans might be seen as having died due to C19 from May 2020 until May 2021, encompassing the ‘second and third Corona waves’. In the interval from May 2021 until May 2022, another 23 348 Germans might be seen as having died due to C19. The total annual NAA-conditional EMCs from May 2020 until May 2022 add up to exactly 37 427 + 23 348 = 60 775; see the ‘excess’ in the last rows of the last but one and third from last column of tables 3 and 5, respectively. As another side note, this differs by less than 1400 from the NAA-conditional EMC of 27 176 + 32 245 = 59 421 for the roughly five-months-earlier 2-year period covering exactly 2020 and 2021. Hence, in Germany, between March 2020 and mid-May 2022, no more than 66 700 persons may have died due to C19 (NAA-conditional EMC), including between 5650 and 5920 excess (due to C19) deaths during the ‘first Corona wave’ in March/April 2020. The latter numbers have been estimated from [40] and [41], respectively. In [40], they calculated an all-cause EMC of 8071, with their mean of 2016–2019 AM baselines being lower than ours averaged over 2014–2019; furthermore, their number (8674) of ‘C19 deaths’ according to the RKI multiplied by 0.65 (excess(total)/*D*_PF_ in tables 4 and 6) is about 5650; official RKI data about a year later [41] deviated slightly: 9104 ‘C19 deaths’, and thus an estimated number of 5920 excess (due to C19) deaths. Another view on figure 1 supports these statements: the strong influenza seasons in 2002/03, 2012/13 and 2014/15 show comparable patterns to the flu season 2020/21 ‘Corona wave’.

Further resolving tables 3 and 5 (entire years) into age groups reveals that NAA-conditional deaths are almost exclusively attributable to the age groups 60+. Within the age group of 50–59 years, only 300 + 340 = 640 fatalities within two years may be ascribed to C19, and for age groups below 50, the negative annual excess numbers would be logged as ‘overall zero deaths due to C19’. In light of this, the 141 + 153 = 294 deaths ‘due to C19’ during the two ‘Corona pandemic’ flu seasons (tables 4 and 6) in the age group 40–49 can be interpreted as people who must have had pre-conditions to die anyway within the next half of a year.

## 4. Discussion

### 4.1. Using standardized mortality ratios to distinguish between deaths ‘with’ and ‘due to’ C19

We have presented and evaluated in §§2.4 and 3.3, respectively, a method to distinguish between concomitant (with) and causative (due to) C19 deaths. In brief, we calculated the expected AMC within the cohort of NAA-positive subjects (*D*_AM|PF_), using our validated model (see further discussion in §4.3), and subtracted this value from the number of observed deaths within this cohort (*D*_PF_) to obtain the excess value. These excess deaths can be assumed to have died ‘due to’ C19, whereas subjects in *D*_PF_ can only be labelled as having died ‘with’ C19. Another way of representing the ratio of deaths ‘due to’ C19 (excess/*D*_PF_) is using the standardized mortality ratio (SMR; symbol *σ*_PF_) as follows:

4.1 $$\sigma_{\mathrm{PF}}=\frac{D_{\mathrm{PF}}}{D_{\mathrm{AM}|\mathrm{PF}}}=\frac{D_{\mathrm{PF}}}{D_{\mathrm{PF}}-\text{excess}}=\frac{1}{1-(\text{excess}/D_{\mathrm{PF}})}\;⟺\;\frac{\text{excess}}{D_{\mathrm{PF}}}=\frac{1}{1-\sigma_{\mathrm{PF}}}.$$

In the following, we will compare our calculated SMR values, given in tables 3–6, to the literature values.

Von Stillfried *et al.* [41] conducted autopsies of Germans clinically suspected to have been struck by C19 and deceased after having received a positive NAA or antigen test (within five weeks in about 90% of the cases). They claimed that in 86% of the autopsies conducted on 986 people, C19 was the *underlying* cause of death according to pathological evidence. However, according to [41, fig. 1], the authors had initially excluded autopsy results of 87 people with ‘unspecific cause of death’ written in their final autopsy report, and 22 persons with the report status ‘cause of death pending’. Thus, basic population accounted for 1095 fatalities that either entered the autopsy procedures with a positive PCR or antigen test, or were at least tested positively post-mortem (‘… test … either pre-clinical, clinical or at post-mortem’ [41]). Consequently, the 86% ‘C19-is-the-underlying-cause’ fraction has been in fact a 77% fraction ($0.77=0.86\cdot \frac{986}{1095}$). With almost 90% of the dead examined in [41] having been of age 60–90 years, we can directly compare their actual fraction value 77% to our estimated fraction of NAA-conditional excess deaths in the same age cohort during the flu season 2020/21 (table 4). We find $\text{excess}/D_{\mathrm{PF}}=\frac{\text{45 065}}{\text{59 434}}=0.76$, i.e. a (percentage) fraction of 76% ‘deaths due to C19’ in these old cohorts, which is remarkably close to the 77% provided by [41]. The corresponding SMR is $\sigma_{\mathrm{PF}}=\frac{1}{1-0.76}=4.17$ according to our estimation; 77% would correspond to *σ*_PF_ = 4.34. During the *whole-year* interval May 2020–May 2021 (table 3), when compared with ‘*wave*’ or *flu-seasonal* intervals, respectively, our estimated fraction in this 60-to-90-years age cohort is lower, namely 61%, which corresponds to $\sigma_{\mathrm{PF}}=\frac{1}{1-0.61}=2.56$. Generally, the *σ*_PF_ values did practically not change for the succeeding (2021/22) flu-seasonal and whole-year intervals, respectively.

The SMR or ‘due-to-C19’ fraction, equivalently, can further be compared to the ‘index of manifestation (of C19)’ *μ*_C19_ (‘Manifestationsindex’ [42]), which the RKI proposed to be the fraction of those actually showing symptoms of C19 among all those having received a positive NAA test; the RKI gives a range (across all age cohorts) *μ*_C19_ = 0.55 … 0.85 [42]; they had extracted these values from the literature until 29 November 2021. More recent sources have provided the following values: 59.5% [43], 35–86% [44] (interquartile ranges) and 56% [45]. Now applying the logic that a person can only die of C19 if C19 symptoms have become manifest, these *μ*_C19_ values can be directly compared to the ‘due-to-C19’ fraction (or the SMR): our estimated values $(1-(1/\sigma_{\mathrm{PF}}))\cdot 100\%$ across all age cohorts are 46% (table 3) and 45% (table 5) for the whole-year intervals, and 66% (table 4) and 65% (table 6) for the flu season intervals. Altogether, our calculations of the fraction or SMR, respectively, well match data from autopsies [41], the RKI [42] and the literature, respectively.

### 4.2. Synopsis of our estimates of all-cause and NAA-conditional EMCs in the light of otherwise reported values

We suggest that our *NAA-conditional* EMC, which is calculated from RKI and Destatis data only, represents the very number of persons who have died *due to* C19. For Germany, we estimate that 59 421 persons have died due to C19 during the two complete 2-year period 2020 and 2021. This total NAA-conditional EMC accounts for roughly 50% of the 115 537 deaths officially counted by the RKI during this 2-year period as *related to* C19 [20] (2020: 44 238; 2021: 71 299; 2022: 48 499). However, we found even a *negative* total *all-cause* EMC of −11 479 (resting solely upon Destatis data; table 1) in those two years; a minor EMC of 6992 in 2021 and moderate under-mortality of −18 471 in 2020. The year 2022 has brought an overall German all-cause EMC of 42 210 fatalities, which is the only annual signal significantly beyond the 90% CI (except from an under-mortality in 2019). This finding is not discussed here, as the issue of all-cause EM revolved for age cohorts is going to be dealt with in a follow-up study.

The correspondingly estimated all-cause EMCs for the flu seasons (table 2) 2020/21 and 2021/22 have shown values of 27 594 and 31 123, respectively, which can be characterized as two typical, moderately severe flu seasons having occurred back to back. They followed however two essentially skipped flu seasons 2018/19 und 2019/20, with EMCs of −22 518 and −22 041, respectively. The comparison of annual with flu-seasonal AMCs implies that deaths occurring during these flu seasons were most likely to be due within the next months anyway. This view is strengthened by the observation of practically all of those deceased due to C19 being older than 60 years (tables 3–6), accepting that all-cause EMCs were dominated by NAA-conditional EMCs (52 738 and 30 099, respectively), and the NAA-conditionally selected sub-group reflecting a sub-population of lower health on average. Further note that the all-cause EMC values (tables 1 and 2) calculated by our simplest, constant AMC model are even less ‘dramatic’ than by the exponential one, when referring to the flu seasons in particular. As an additional model validation, the ‘flu deaths’ reported by the RKI between 2000/01 and 2017/18 (last column in table 2) consistently match our seasons of calculated under-mortality (RKI: zero flu deaths) and the magnitudes within seasons of calculated all-cause EM. The high consistency of both our model estimates strengthens the evidence of exactly non-exceptional ‘Corona pandemic’ mortality in Germany. The high preliminary all-cause EMC of 49 563 within the season 2022/23, which is likely to moderately increase due to delayed registration, will likewise be discussed within a follow-up, age-cohort-resolved study.

Exceptionally deadly population dynamics in accordance with the WHO’s before-2009 description of what should be classified as an ongoing pandemic (in 2003–2009 comprising the qualifier ‘… resulting in epidemics world-wide with enormous numbers of deaths and illness’) [46–48] are not discernible for the years 2020 and 2021 in figures 4, 5 and tables 1, 2. Hence, data contradict a classification of C19 as an ‘exceptionally severe disease’ [49] or declaring its spread as a ‘public health emergency of international concern’ [50].

On 5 May 2022, the WHO report announced a *calculated* mean of 66 794 ‘C19-associated’ excess deaths for 2020, and 128 194 for 2021 [19], i.e. a sum of 194 988 EMC. Due to apparent ‘data/model issues’ [15], these arguably pandemic-worthy numbers were subsequently lowered to 122 432 (=55 648 + 66 784) [15,18]. The currently available WHO dataset as of 19 May 2023, however, contains a sum of merely 101 505 (=34 514 + 66 991), without any publication reference. This last number even under-quotes the RKI count of 115 537. We found another three articles reporting all-cause EMCs for Germany between 2020 and 2021, which differed by a full order of magnitude; first, a two-paper study from German and Swedish scientists [51,52] who proposed a total EMC of 6317 + 23 399 = 29 716; second, an article by German scientists [53] who used a similar method and proposed a total EMC of 4015 + 33 980 = 37 995; third, an article by the ‘COVID-19 Excess Mortality Collaborators’ [54], funded by the ‘Bill & Melinda Gates Foundation’, who claimed the highest EMC, namely, a mean 203 000 within a narrow range from 193 000 to 210 000. Note that the latter source, although calculating *all-cause* EMC, denominate this value exclusively ‘due to COVID-19’.

What follows is a critical review of these vastly different EMC estimates and their underlying modelling procedures.

### 4.3. Shortfalls and consequences for modelling reliable EMC

At the end of the previous section, we have found that four mathematical models found four inherently different all-cause EMCs for Germany between 2020 and 2021: from −11 500 (our study) to 30 000–38 000 [51–53] to 122 000 [15,18] (with them having reduced from initially 195 000 [19] published in the first WHO report) to 203 000 [54]. In this section, we aim at resolving how these different estimates arose from a prima facie unambiguous dataset of a German AMC. Note that these EMCs are independent of any NAA-conditional counts and should thus not be equated or confused with deaths ‘due to C19’ (cf. §3.3), but thoroughly scrutinized. Three crucial model constituents are juxtaposed:

(1) The data basis, including the range of years considered.
(2) Model equations and (over-)parameterization.
(3) Age cohort distribution and demographic changes.

(*Ad 1*) We compare the underlying datasets of all four models. As stated in §2.2, we used German AMC data of 2000–2019 (exponential model) and 2014–2019 (constant model), respectively, as an input taken from Destatis [14,27]. The Wang *et al.* model [54] took the same Destatis data, but of 2016–2019 only [14]. De Nicola *et al.* [51,52] as well as Kuhbandner & Reitzner [53] likewise used Destatis data, but life tables (annual AMRs instead of AMCs) of 2017–2019 [55]. The WHO-endorsed models [15,18] relied on AMCs of 2015–2019 taken from the Human Mortality Database [56]. While the different datasets should in principle comprise the same (or at least comparable) AMC data, all models were based on different time intervals. A look at figure 5 suggests that this choice may well determine the reliability of model outputs. While 2014 was a year of rather low AM, 2015 was above the AM expectation compared to our exponential model, 2016–2018 showed a little below-average mortality, and 2019 was a year of significant under-mortality. Now, the fewer the years before 2020 considered in any kind of fitting, smoothing, or superposing, the higher the impact of the outlier 2019, i.e. the trend to generally under-estimate AM-model-based *expectations* and thus report over-estimated EMCs. In the extreme case of choosing the interval 2015–2019 and taking weekly counts (figure 4), a mere linear fit of annual AMCs might well lead to even a slightly negative overall trend of prognosticated AMCs, if not considering the demographic changes (see below). Tracing the effect on our constant model, we estimated in table 1 an under-mortality in 2020 and 2021 combined of −4383 persons when using a six-years AMR average (2014–2019). If we switched to 4 years (2016–2019) or 3 years (2017–2019) averaging, we would end up with EMCs of 11 976 or 15 268, respectively. Using the 4 years median as suggested by Destatis [33], we end up with a 2 years EMC of 22 354 ( = −595 + 22 949); see table 1. As can be seen in figure 4, significant outliers such as 2019 have greater influence on the 4 years model than on our 6 years model. Also, constant AMR models work more reliably in regions of constant AMR and fail to prognosticate the situation in years with changing AMR; see the Destatis-model-based estimates for 2004–2012. This should be taken into account when using this method to estimate other pandemic key figures—such as the basic reproduction number [57]—based on EMC.

(*Ad 2*) We sketch the underlying models. The WHO-endorsed models [15,18] and the Wang *et al.* model [54] employed a Poisson sampling approach to calculate estimated AMCs for 2020 and 2021 on the basis of their respective datasets. Both approaches combined the observed AM fluctuations around the sampled mean with spline-based tends to take account of annual as well as seasonal AM changes. While the Wang *et al.* model [54] used linear splines for seasonal changes and cubic splines for yearly (‘secular’) trends, the WHO model in its initial form [16] used cubic splines for seasonal changes and thin-plate splines for yearly trends. Due to the German and Swedish excess estimates in [16] having initially been ‘too high’ [15], the cubic-spline part was changed in the WHO-endorsed models [15,18] to thenceforth employ linear trends for yearly changes in Germany. What is more in the Wang *et al.* model [54], the placement of the last knot for their splines was varied in order to yield four different extrapolations. With the implementation of another two sub-models, a Poisson regression model part and a ‘last-year’ (2019) model part, the Wang et al. model finally consists of a weighted mean of six sub-models. In the De Nicola *et al.* model [51], life tables (AMR) are directly converted into expected AMCs, while using three differently detailed approaches (see next paragraph). Thereby, the years 2017–2019 contributed equally to forming the prognosis for 2020, for both yearly and weekly data, with only applying the most elaborated of the three approaches. The latter approach was also used in Kuhbandner & Reitzner [53], with additionally distinguishing members of the population into males and females. For an immediate comparison, we recall our model approach to calculate an expected AMC: we estimate age-cohort-specific AMRs of 2020 and 2021 by extrapolating exponential fits to the AMR courses between 2000 and 2019 (or, alternatively, constant fits between 2014 and 2019), multiply these extrapolated AMRs by their corresponding proportions of the demographic distribution as prognosticated by Destatis [29], and sum all so-weighted cohorts’ AMCs.

By looking at the required parameters, we find that the De Nicola *et al.* [51,52] as well as the Kuhbandner & Reitzner [53] model required no additional parameters, but rather assumptions on how life tables were to convert to future predictions of AMC. Our two model alternatives required either two (exponential) or one (constant) fit parameters per each of the eight age cohorts, together with each cohort’s AMR reference value in 2000 as a non-fit parameter. We emphasize that both formulations arose from a careful look at the normalized AMR courses over the twenty ‘pre-pandemic’ years (figure 2*b*) and may not be applicable to the situation in other countries. Other plain, elementary, or special functions may yet be well suitable. For the WHO-endorsed models [15,18] or the Wang *et al.* model [54], we find no definite number of required parameters. However, as cubic splines require at least four parameters more than there are control (data) points, plus parameters for the sampling, plus additional sub-models in the Wang *et al.* model, an investigation into their Akaike information criterion values would be required to avoid over-fitting [58]. While the Poisson sampling method has the general advantage of being flexibly applicable in cases of data transfer from countries being incomplete, the predictions for future developments have to be taken with extreme caution (see appendix C). Wang *et al.* correctly state [54, suppl.] that cubic-spline extrapolation is subject to large uncertainties. This may have been the reason for the WHO-endorsed models eventually using linear regression instead of splines [15,18]. In any case, the low AMC observed in 2019 seems to have driven the severe under-estimation of 2020 and 2021 AMCs and hence severe over-estimations of EMC (see the first model constituent above).

(*Ad 3*) Now turning to the third crucial model constituent, we showed in §3.1 that age cohort distribution and demographic changes play a crucial role in the prediction of future AMCs. Evidently, a Simpson’s paradox yields an increasing average AMR since 2000, while the trend is decreasing within each age cohort. Our AMC model as well as the De Nicola *et al.* and Kuhbandner & Reitzner models [51,53] included the demographic changes in Germany, the latter two additionally including age cohort transitions within years (Lexis diagram). The WHO-endorsed models [15,18] do not include age cohort resolution, but provide *a posteriori* calculations of deaths within the age cohorts. The Wang *et al.* model [54] contains neither age cohort nor demographic considerations at all.

Summarizing, our model produced the least deviations between modelled and measured AMCs, or, in other words, the least absolute EMCs. This had been achieved by (i) using a suitable long data history, (ii) assessing the Germany-specific time course of all age cohorts’ AMRs, (iii) using a low-parametric and straightforward model and (iv) accounting for time-dependent age cohort distribution and thus demographic effects. In the past, the RKI had introduced similar model calculations [59], also applied to estimate flu-seasonal EMCs [60], and latest to the 2017/18 season [30, pp. 13,17] (table 2); but up to date any EMC estimation by the RKI during the ‘Corona pandemic’ period is owing.

The urgent need for reliable, transparent and comprehensible estimates of *all-cause* EMCs on the basis of all-cause AMCs holds in particular, as all of the above WHO-associated publications [15,18,54] relate EMC to solely C19. Furthermore, no distinction in deaths ‘due to C19’ and ‘with C19’ in the light of NAA-conditional mortality (cf. §3.3) has been made as of yet. The herein presented methodology may provide a blueprint for other countries and serve as a benchmark in Germany for both demands.

## 5. Conclusion

World-wide media coverage of the ‘CoViD-19 pandemic’ is unsurpassed by any event recently (or probably ever). Accordingly, a plethora of data have been collected and analysed to yield information and statistical knowledge about infection dynamics, as well as key performance indicators for their containment. The dramatic number of increased mortality was one of the most important arguments by politicians to impose strict socio-economical measures against the population. In this work, we aimed for scrutinizing the reportedly high EM during 2020 and 2021. In stark contrast to the WHO estimates of 101 500–195 000 excess deaths in Germany in those 2 years, we even found a net under-mortality of −11 500, which implies that nothing but ‘dying as usual’ has happened, at least, when looking at the net proceedings across all age cohorts. Our analysis shows that the WHO estimates are an exaggeration, which arises from (i) a short-termed data basis, (ii) error-prone spline extrapolations and (iii) missing age-cohort-specific all-cause mortality characteristics, cloaking an underlying Simpson’s paradox. Further, all WHO-estimated excess deaths are attributed to solely C19. Here, we have suggested a clear criterion to distinguish, at least on the epidemiological level, between deaths caused ‘due to C19’ or only accompanied ‘with C19’. Based on a thorough analysis of NAA-conditional mortality, out of the about 115 000 ‘C19 deaths’ proclaimed by the RKI for 2020 and 2021 together, only about 59 000 or roughly 50% may be considered causatively related to C19.

Several open questions arise from our analysis, requiring thorough subsequent investigation: do estimates of EM for other countries likewise suffer from ignoring demographic developments? How could the exaggerated, non-validated EMC calculations under the auspices of the WHO become accepted [61] on a large scale? Should not political measures undergo reassessment in light of realistic and reliable EM counts now being available? Why have no calculations of the kind presented here been conducted by German authorities as of mid-2023, at least?

## Figures and Tables

**Figure 6.**
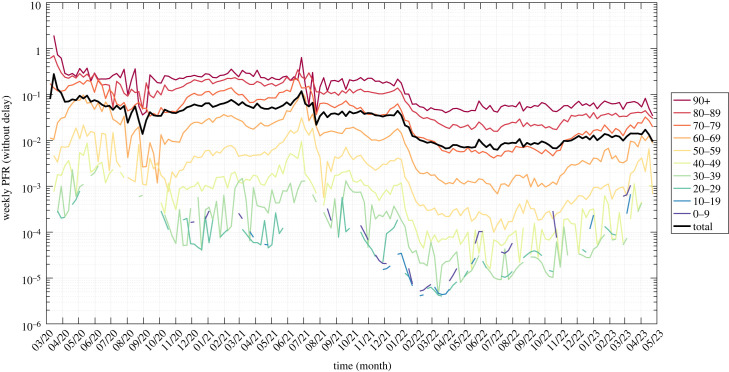
For each German age cohort, the raw, week-resolved PFR values in the period 05/2020 to 07/2022, i.e. weekly ratios *D*_PF_/NAA_pos_ of the NAA-conditional (C19) death counts *D*_PF_ to the number NAA_pos_ of NAA-positive tests, as published by German authorities.

**Figure 9.**
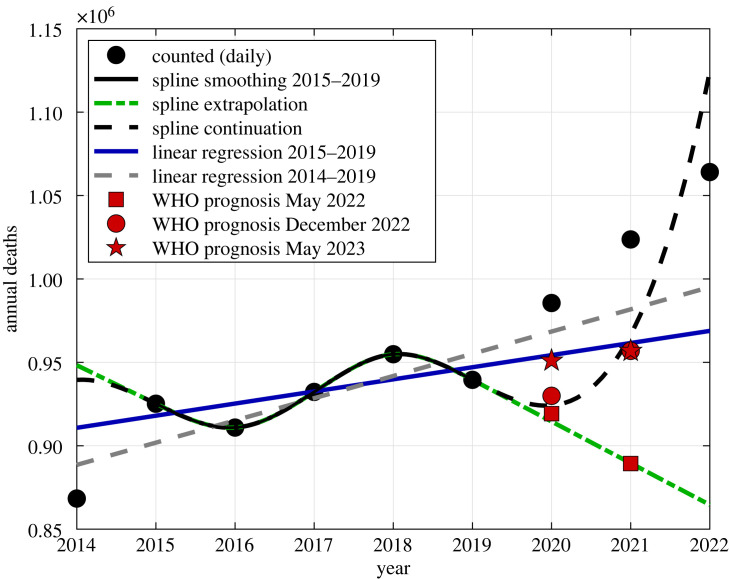
Four possible ways to extrapolate the annual death counts in Germany between 2015 and 2019. Firstly and secondly, using a smoothing spline (black line), its extrapolation (green dash-dotted line) or its continuation (black dashed line) can be calculated. Thirdly, a linear regression (blue line) on the basis of 2015–2019 is conducted. These three methods well match the prognoses given by the WHO in December 2022 (red circles), May 2022 (red squares), and May 2023 (red stars), respectively. Note that for 2021, red circle and star overlay. Fourthly, for comparison, a linear regression (grey dashed line) on the basis of 2014–2019 is shown.

## Data Availability

Supplementary material: A German version of title and abstract is available on FigShare [63].

## Appendix A. The course of the PFR during the SARS-CoV-2 era: delay, tests and persons

The results presented in this appendix are based on the calculations introduced in §2.4. In particular, we calculated a general time delay for all age cohorts from the time of tested infection to (possible) death. Figure 6 shows the time courses of the age cohorts’ (raw) weekly PFR values (*D*_PF_/NAA_pos_) during the period of (putatively only) SARS-CoV-2 infections, as calculated by dividing the number of C19 deaths *D*_PF_ per week by the number of positive NAA tests NAA_pos_ per the same week. Next, figure 7 shows the mean of PFR values (*D*_PF_/NAA_pos_) per age cohort when shifting the course of the registered death counts *D*_PF_ by up to 5 weeks forward in time with respect to course of the registered counts of positive tests NAA_pos_. For each age cohort, we see that the PFR attains a minimum between 4- and 24-day shifts (leaving away those younger than 20 years). For both simplicity as well as clarity reasons and because, on the one hand, almost all minima were very flat, and, on the other hand, another study [34] calculated a C19-‘case’-death delay of 13 days in Germany for those older than 60 years, around which our values of all older than 50 years scatter (figure 7), we generally chose a two-week delay, which results in the weekly PFR values displayed in figure 8.

Two things strike to the eye in figure 8. First, there were events of sudden PFR dipping by about an order of magnitude in early October 2020 in the two cohorts of those older than 80 years, each preceded by a peak in mid-September. This is presumably an artefact simply due to very low numbers of both fatalities and NAA-positive persons. Second, after a similar peak–dip event between the end of July and late August 2021, the PFR values generally started to drop, first just slightly since October, and then the drop becoming particularly evident since the start of December 2021.

The effect of the PFR values in late 2021, even more in 2022, and in the flu season 2021/22 being so clearly lower than in 2020 and the season 2020/21, respectively, becomes evident in table 7 in which arithmetic mean values (annual and seasonal) of the fluctuating time courses in figure 8 are given. Our suggestion to interpret such generally and significantly dropping PFR values is that there has been a near one-to-one relation between ‘case’ counts (NAA_pos_) and positively tested persons *only until* summer or maybe autumn 2021. Afterwards, multiple NAA testings per person and year must have become a rule. We picked up on this issue in §3.3 in which we used the interpretation of multiple testings to implement an additional NAA_pos_ data treatment that infers processed PFR values for 2021 and 2021/22, which prove absolutely reasonable.

In table 7, we have juxtaposed PFR and AMR values (annual and flu-seasonal), *r*_PF_ and *r*_AM_, respectively, for the years 2019–2021 and the flu seasons from 2019/20 to 2021/22, respectively. The AMR values of the flu seasons were scaled by 52/33 (33 weeks per flu season), symbolized by *r*_AM,*f*_*, to make them comparable to both the annual AMR and the PFR values. Interesting observations in table 7 are summarized in the following: the first three items are about AM; the subsequent ones compare AM (*r*_AM_, *r*_AM,*f*_*) and NAA-conditional death rates (*r*_PF_), that is, the likelihoods of a German citizen to die within a year (or flu season scaled to a year, respectively) due to any cause (unconditional: *r*_AM_ or *r*_AM,*f*_*, respectively) and to die within the respective interval (year or flu season, respectively) after having been tested positively in a NAA test (conditional: PFR). Due to the reasons given in the preceding paragraph, PFR values of 2021 and the flu season 2021/22 are not addressed here.

— All AMR values slightly increasing from 2019 to 2021 are fully consistent with the correspondingly increasing total EMC values evident from table 1. The AMR values in 2020 and 2021 were higher than in 2019 for those with the highest AMR values (90+ and 80–89 cohorts), only slightly higher down to the age of 30, and even minutely lower for the 0–29 cohort.
— The ${\mathrm{AMR}}_{\;f}^{*}$ values of the flu (second and third Corona waves) season 2020/21 are, in all cohorts 60+, moderately higher than in the flu season 2019/2020, slightly higher for the 30–59 cohort, and even minutely lower for the 0–29 cohort. The very same holds for the classification of the flu season 2020/21 ${\mathrm{AMR}}_{\;f}^{*}$ when compared with the annual AMR values both in 2020 and in 2021. This, most notably, owes to the fact that the flu season 2019/20 was exceptionally mild (see again the total excess values in table 2), with its ${\mathrm{AMR}}_{\;f}^{*}$ values being in any cohort even lower than the annual AMR values in all 2019–2021, except for the extraordinary low 2019 AMR in the 80+ cohorts: not only was the flu season 2019/20 very mild, but also the preceding year 2019 was obviously extraordinarily mild (see again table 1 indicating a marked total under-mortality, negative EMC, in 2019).
— The 2021/22 ${\mathrm{AMR}}_{\;f}^{*}$ values are practically the same as in the season 2020/21, across all cohorts, which is again fully consistent with the respective EMC values being almost the same in these seasons, according to table 2.
— In 2020, the PFR value of the oldest (90+: 0.2945) is higher than all AMR and ${\mathrm{AMR}}_{\;f}^{*}$ values in 2019–2021 and 2019/20–2021/2022, respectively. Interestingly, the PFR value in the flu season 2020/21 was lower than in the year 2020, and even lower than the respective ${\mathrm{AMR}}_{\;f}^{*}$ values in both flu seasons 2020/21 and 2021/22.
— The PFR values of the three cohorts 60–89 were all between two and three and a half times higher than the respective AMR values in 2019 and 2020, and ${\mathrm{AMR}}_{\;f}^{*}$ values in the flu season 2020/21, respectively. Again interestingly, like in the 90+ cohort, the PFR values of these three cohorts were lower in the flu season 2020/21 than in the year 2020.
— For the 50–59 cohort, the PFR values in 2020 and the flu season 2020/21 were just minutely higher than the respective AMR and ${\mathrm{AMR}}_{\;f}^{*}$ values. For all younger cohorts, the PFR values in both intervals were clearly lower (only a fourth among the youngest) than all AMR values from 2019 to 2021 and the ${\mathrm{AMR}}_{\;f}^{*}$ values of the flu seasons 2020/21 and 2021/22.
— The *total* (weighted by cohort proportion) PFR values in Germany were roughly double as high as total German AMR values, as they were dominated by the NAA-conditional death rate excess (PFR > *AMR*) of the four 60+ cohorts in 2020, and the three 60-89 cohorts in the flu season 2020/2021 ($\mathrm{PFR}>{\mathrm{AMR}}_{\;f}^{*}$).

**Table 7.** Calculated mean German AMR and PFR values (*r*_AM_ and *r*_PF_, respectively) from 2019 to 2021, including the flu seasons 2019/20, 2020/21 and 2021/22.

	year						flu season					
	2019		2020		2021		2019/20		2020/21		2021/22	
age cohort	AMR	PFR	AMR	PFR	AMR	PFR	${\mathrm{AMR}}_{\;f}^{*}$	PFR	${\mathrm{AMR}}_{\;f}^{*}$	PFR	${\mathrm{AMR}}_{\;f}^{*}$	PFR
90+	0.2286	—	0.2401	0.2945	0.2417	0.2174	0.2331	—	0.2668	0.2544	0.2637	0.07082
80–89	0.07219	—	0.07440	0.2168	0.07529	0.1491	0.07238	—	0.08320	0.1875	0.08116	0.03963
70–79	0.02678	—	0.02697	0.1016	0.02756	0.06855	0.02650	—	0.02981	0.09244	0.02810	0.01343
60–69	0.01086	—	0.01098	0.02420	0.01148	0.01875	0.01072	—	0.01200	0.02322	0.01178	3.197 × 10^−3^
50–59	4.221 × 10^−3^	—	4.280 × 10^−3^	4.455 × 10^−3^	4.510 × 10^−3^	4.474 × 10^−3^	4.158 × 10^−3^	—	4.649 × 10^−3^	4.739 × 10^−3^	4.566 × 10^−3^	7.158 × 10^−4^
40–49	1.521 × 10^−3^	—	1.527 × 10^−3^	1.120 × 10^−3^	1.598 × 10^−3^	1.186 × 10^−3^	1.498 × 10^−3^	—	1.623 × 10^−3^	1.233 × 10^−3^	1.618 × 10^−3^	1.879 × 10^−4^
30–39	6.021 × 10^−4^	—	6.063 × 10^−4^	2.601 × 10^−4^	6.201 × 10^−4^	3.904 × 10^−4^	5.957 × 10^−4^	—	6.290 × 10^−4^	3.590 × 10^−4^	6.404 × 10^−4^	6.010 × 10^−5^
0–29	3.005 × 10^−4^	—	2.861 × 10^−4^	7.309 × 10^−5^	2.929 × 10^−4^	4.695 × 10^−5^	2.814 × 10^−4^	—	2.804 × 10^−4^	6.137 × 10^−5^	2.975 × 10^−4^	8.203 × 10^−6^
total	0.01126	—	0.01179	0.02903	0.01221	0.01344	0.01137	—	0.01315	0.02460	0.01295	2.111 × 10^−3^

## Appendix B. Optimal fit parameters

See tables 8 and 9.

**Table 8.** Annual AMRs plus CIs, calculated by both the constant and the exponential model; see §2.3. For the exponential model, function parameters are given as well. ${\;}^{†}$For the age cohort 90+, a constant instead of an exponential fit was conducted.

	constant model		exponential model			
	AMR 2014–2022		function parameters		AMR 2020–2022	
age cohort	mean	CI	*r*_AM,coh_(2000)	$\left(\begin{array}{c} a_{\mathrm{coh}}\\ b_{\mathrm{coh}} \end{array}\right)$	mean	CIs
0–29	3.113 × 10^−4^	[2.929 × 10^−4^; 3.297 × 10^−4^]	4.813 × 10^−4^	$\left(\begin{array}{c} 0.1237\\ 0.5927 \end{array}\right)$	3.017 × 10^−4^	[2.867 × 10^−4^; 3.168 × 10^−4^]
					2.998 × 10^−4^	[2.846 × 10^−4^; 3.151 × 10^−4^]
					2.981 × 10^−4^	[2.826 × 10^−4^; 3.136 × 10^−4^]
30–39	6.135 × 10^−4^	[5.897 × 10^−4^; 6.372 × 10^−4^]	9.015 × 10^−4^	$\left(\begin{array}{c} 0.1148\\ 0.6162 \end{array}\right)$	5.903 × 10^−4^	[5.609 × 10^−4^; 6.196 × 10^−4^]
					5.865 × 10^−4^	[5.566 × 10^−4^; 6.164 × 10^−4^]
					5.831 × 10^−4^	[5.528 × 10^−4^; 6.135 × 10^−4^]
40–49	1.620 × 10^−3^	[1.479 × 10^−3^; 1.762 × 10^−3^]	2.376 × 10^−3^	$\left(\begin{array}{c} 0.07947\\ 0.5599 \end{array}\right)$	1.544 × 10^−3^	[1.485 × 10^−3^; 1.603 × 10^−3^]
					1.528 × 10^−3^	[1.467 × 10^−3^; 1.588 × 10^−3^]
					1.513 × 10^−3^	[1.450 × 10^−3^; 1.575 × 10^−3^]
50–59	4.399 × 10^−3^	[4.143 × 10^−3^; 4.655 × 10^−3^]	5.809 × 10^−3^	$\left(\begin{array}{c} 0.07299\\ 0.6587 \end{array}\right)$	4.287 × 10^−3^	[4.138 × 10^−3^; 4.436 × 10^−3^]
					4.255 × 10^−3^	[4.101 × 10^−3^; 4.408 × 10^−3^]
					4.224 × 10^−3^	[4.066 × 10^−3^; 4.382 × 10^−3^]
60–69	0.0109	[0.01054; 0.01127]	0.0131	$\left(\begin{array}{c} 0.08945\\ 0.7829 \end{array}\right)$	0.01073	[0.01037; 0.0111]
					0.01069	[0.01032; 0.01106]
					0.01065	[0.01027; 0.01103]
70–79	0.02702	[0.02562; 0.02843]	0.03464	$\left(\begin{array}{c} 0.1369\\ 0.7365 \end{array}\right)$	0.0261	[0.02408; 0.02812]
					0.02603	[0.02398; 0.02807]
					0.02596	[0.02389; 0.02803]
80–89	0.07753	[0.07003; 0.08504]	0.09886	$\left(\begin{array}{c} 0.1881\\ 0.7944 \end{array}\right)$	0.07901	[0.07233; 0.08569]
					0.07893	[0.0722; 0.08565]
					0.07886	[0.0721; 0.08562]
90+	0.2316	[0.2167; 0.2464]	0.2825	0.8555${\;}^{†}$	0.2417	[0.2131; 0.2702]

**Table 9.** Flu seasonal AMRs plus CIs, calculated by both the constant and the exponential model; see §2.3. For the exponential model, function parameters are given as well. ${\;}^{†}$For the age cohort 90+, a constant instead of an exponential fit was conducted.

	constant model		exponential model			
	AMR 2014/15–2022/23		function parameters		AMR 2020/21–2022/23	
age cohort	mean	CI	*r*_AM,coh_(00/01)	$\left(\begin{array}{c} a_{\mathrm{coh}}\\ b_{\mathrm{coh}} \end{array}\right)$	mean	CIs
0–29	1.956 × 10^−4^	[1.737 × 10^−4^; 2.175 × 10^−4^]	2.939 × 10^−4^	$\left(\begin{array}{c} 0.123\\ 0.6102 \end{array}\right)$	1.891 × 10^−4^	[1.821 × 10^−4^; 1.961 × 10^−4^]
					1.880 × 10^−4^	[1.808 × 10^−4^; 1.951 × 10^−4^]
					1.870 × 10^−4^	[1.797 × 10^−4^; 1.942 × 10^−4^]
30–39	3.918 × 10^−4^	[3.594 × 10^−4^; 4.243 × 10^−4^]	5.598 × 10^−4^	$\left(\begin{array}{c} 0.1289\\ 0.6474 \end{array}\right)$	3.774 × 10^−4^	[3.606 × 10^−4^; 3.942 × 10^−4^]
					3.756 × 10^−4^	[3.586 × 10^−4^; 3.926 × 10^−4^]
					3.740 × 10^−4^	[3.567 × 10^−4^; 3.913 × 10^−4^]
40–49	1.030 × 10^−3^	[9.042 × 10^−4^; 1.155 × 10^−3^]	1.478 × 10^−3^	$\left(\begin{array}{c} 0.06265\\ 0.5193 \end{array}\right)$	9.707 × 10^−4^	[9.301 × 10^−4^; 1.011 × 10^−3^]
					9.583 × 10^−4^	[9.165 × 10^−4^; 1.000 × 10^−3^]
					9.468 × 10^−4^	[9.034 × 10^−4^; 9.901 × 10^−4^]
50–59	2.828 × 10^−3^	[2.566 × 10^−3^; 3.091 × 10^−3^]	3.634 × 10^−3^	$\left(\begin{array}{c} 0.03195\\ 0.458 \end{array}\right)$	2.704 × 10^−3^	[2.608 × 10^−3^; 2.801 × 10^−3^]
					2.671 × 10^−3^	[2.570 × 10^−3^; 2.772 × 10^−3^]
					2.640 × 10^−3^	[2.534 × 10^−3^; 2.746 × 10^−3^]
60–69	7.110 × 10^−3^	[6.663 × 10^−3^; 7.556 × 10^−3^]	8.178 × 10^−3^	$\left(\begin{array}{c} 0.03663\\ 0.6993 \end{array}\right)$	6.901 × 10^−3^	[6.659 × 10^−3^; 7.143 × 10^−3^]
					6.858 × 10^−3^	[6.605 × 10^−3^; 7.112 × 10^−3^]
					6.818 × 10^−3^	[6.552 × 10^−3^; 7.083 × 10^−3^]
70–79	0.01776	[0.01646; 0.01906]	0.02207	$\left(\begin{array}{c} 0.1359\\ 0.7598 \end{array}\right)$	0.01712	[0.01621; 0.01802]
					0.01707	[0.01616; 0.01799]
					0.01704	[0.01611; 0.01796]
80–89	0.05113	[0.04352; 0.05873]	0.0622	$\left(\begin{array}{c} 0.03923\\ 0.6318 \end{array}\right)$	0.04975	[0.04624; 0.05326]
					0.04935	[0.04569; 0.053]
					0.04896	[0.04513; 0.05279]
90+	0.1563	[0.1401; 0.1726]	0.1682	0.9554${\;}^{†}$	0.1607	[0.1504; 0.171]

## Appendix C. Numerical extrapolation of death counts

This appendix aims to offer an explanation on how the different WHO-endorsed estimates of annual death counts in 2020 and 2021 [15,18,19] might have been achieved. It is important to note that the underlying generalized additive model (GAM) is a phenomenological one, i.e. does not incorporate structural knowledge about a population—such as size, age distribution or age-related mortality rates—but is solely based on the weekly reported numbers of deaths between 2015 and 2019. Condensing the model by (i) omitting the Poisson sampling, as well as (ii) the within-year variation, and (iii) relying on yearly instead of weekly reports, we want to estimate AMC in 2020 and 2021. The model equation [18, eqn 2] suggests that spline extrapolation was used initially, but that the resulting ‘excess estimate was too high because of a combination of data/model issues’ [15], such that it had eventually been replaced with a linear regression. With the annual AMC of 2015–2019 as a basis, we extrapolate the AMC of 2020 (counted: 985 600) and 2021 (counted: 1 024 000) applying three methods. First, a cubic spline smoothing was conducted using the MatLab routine csaps, with a smoothing parameter of 1, ensuring interpolation. That spline was then extrapolated using the routine fnxtr, with a smoothness condition of order 3. This method’s result well matches the initial (May 2022) WHO prognoses for 2020 (extrapolation: 914 500; WHO: 919 200) and 2021 (extrapolation: 888 600; WHO: 889 300) and would result in a total EMC of roughly 200 000. Second, the natural continuation of the smoothing spline was calculated, i.e. the coefficients from the smoothing spline were used on the enlarged interval until 2021. This method yields values close to the WHO estimate presented in [15,18] for both 2020 (continuation: 924 200; WHO: 929 900) and 2021 (continuation: 956 900; WHO: 963 000), resulting in an EMC of roughly 120 000. Third, the AMC data of 2015–2019 were approximated by a linear function using the routine polyfit with a polynomial of degree 1. The values of this extrapolation are close to those comprised in the latest WHO dataset [19] again for both 2020 (regression: 954 300; WHO: 951 100) and 2021 (regression: 961 600; WHO: 956 900), thus yielding an EMC of around 100 000.

Figure 9 shows the results outlined above. Although all three alternatives constitute vast over-simplifications of the WHO-endorsed models, the congruence is remarkable. This toy example also demonstrates the pitfall of extrapolating a phenomenological GAM, which is formulated as an admonition in the underlying source [58]: ‘extrapolation is still a fairly dangerous thing to do, and we had better not rely on it very far into the future’. Hence, the results of the extrapolation have to be validated—for example by testing the method for previous years—in order to ensure their meaningfulness and to avoid arbitrariness. To demonstrate the latter, an additional fourth extrapolation method, a linear regression that uses the years 2014–2019 as basis, is shown; see also [62]. Here, the values for 2020 (regression: 968 500) and 2021 (regression: 981 800) would result in an EMC of only 59 300, which cuts WHO-endorsed EMC prognoses in half or even thirds, respectively.
